# Inter-species interactions alter antibiotic efficacy in bacterial communities

**DOI:** 10.1038/s41396-021-01130-6

**Published:** 2021-10-09

**Authors:** Michael J. Bottery, Jessica L. Matthews, A. Jamie Wood, Helle Krogh Johansen, Jon W. Pitchford, Ville-Petri Friman

**Affiliations:** 1 Division of Evolution, Infection and Genomics, University of Manchester, Manchester, M13 9PL UK; 2 Department of Biology, University of York, Wentworth Way, York, YO10 5DD UK; 3 Department of Mathematics, University of York, Heslington, York, YO10 5DD UK; 4 The Novo Nordisk Foundation Center for Biosustainability, Technical University of Denmark, Lyngby, Denmark; 5 Department of Clinical Microbiology 9301, Rigshospitalet, Copenhagen, Denmark; 6 Department of Clinical Medicine, Faculty of Health and Medical Sciences, University of Copenhagen, Copenhagen, Denmark

**Keywords:** Antibiotics, Bacterial infection, Microbial ecology

## Abstract

The efficacy of antibiotic treatments targeting polymicrobial communities is not well predicted by conventional in vitro susceptibility testing based on determining minimum inhibitory concentration (MIC) in monocultures. One reason for this is that inter-species interactions can alter the community members’ susceptibility to antibiotics. Here we quantify, and identify mechanisms for, community-modulated changes of efficacy for clinically relevant antibiotics against the pathogen *Pseudomonas aeruginosa* in model cystic fibrosis (CF) lung communities derived from clinical samples. We demonstrate that multi-drug resistant *Stenotrophomonas maltophilia* can provide high levels of antibiotic protection to otherwise sensitive *P. aeruginosa*. Exposure protection to imipenem was provided by chromosomally encoded metallo-β-lactamase that detoxified the environment; protection was dependent upon *S. maltophilia* cell density and was provided by *S. maltophilia* strains isolated from CF sputum, increasing the MIC of *P. aeruginosa* by up to 16-fold. In contrast, the presence of *S. maltophilia* provided no protection against meropenem, another routinely used carbapenem. Mathematical ordinary differential equation modelling shows that the level of exposure protection provided against different carbapenems can be explained by differences in antibiotic efficacy and inactivation rate. Together, these findings reveal that exploitation of pre-occurring antimicrobial resistance, and inter-specific competition, can have large impacts on pathogen antibiotic susceptibility, highlighting the importance of microbial ecology for designing successful antibiotic treatments for multispecies communities.

## Introduction

Antibiotics are a vital tool in the treatment of bacterial infections. However, predicting the outcome of antibiotic treatment of polymicrobial infections is challenging due to the complex and multifaceted inter-microbial and host-pathogen interactions that occur [1]. Identifying pathogens that contribute to disease and antimicrobials that are efficient against them is a key first step to reducing pathogen load [2]. As a result, in vitro antibiotic susceptibility testing using the minimum inhibitory concentration (MIC), the lowest concentration of an antibiotic required to inhibit the growth of a bacterial strain during standardised monocultures [3], is a critical tool to inform decisions on both the choice and dosage of antibiotic interventions. However, MIC testing is not reflective of the true conditions that bacteria face during infection, where the antibiotic susceptibility of a strain can be reduced or magnified by abiotic and biotic factors, i.e., its ecological context. For example, high cell density can significantly reduce the susceptibility of bacteria to antibiotics, referred to as the ‘inoculum effect’ in MIC testing [4–6]. Nutritional environments that more closely resemble the site of infection can also increase MIC of bacterial strains above clinical breakpoints of resistance [7]. Similarly, biofilm formation can profoundly alter MIC measurements due to an increase in tolerance through reduced antibiotic penetration, reduced growth rates and altered stress responses [8–11], leading to alternative susceptibility measures such as minimal biofilm eradication concentration and minimal biofilm inhibitory concentration [12, 13]. Another key limitation of MIC testing is the lack of microbial community context, i.e., the effect of intra- and inter-species interactions within a bacterial community, which could alter the response of its members to antibiotic treatments [14]. As a result, antibiotic sensitivity should be considered as an emergent property in polymicrobial infections determined by both the genetic properties of the pathogen, physical environment—including the host immune system—and the surrounding microbial community [14]. Disregarding this biological complexity could partly explain why conventional susceptibility testing poorly translates to successful clinical outcomes, particularly during the treatment of complex polymicrobial infections [15, 16].

Mounting evidence shows that inter-species interactions within microbial multispecies communities, which are particularly common during polymicrobial infection, can alter the susceptibility of its members to antibiotics [17–19]. These interactions could increase a species’ ability to survive antibiotic treatment if surrounding species confer a protective effect [20]. For example, coculturing members of synthetic fruit fly gut communities led to increases in tolerance to antibiotics in *Acetobacter* species due to physiological responses to changes in pH due to the community composition [19]. The secretion of exoproducts by a community’s members can also alter sensitivity to antibiotic treatments; for example, the secreted *Staphylococcus aureus* streptococcal protein A enhances the biofilm production of *Pseudomonas aeruginosa* leading to increased tobramycin resistance [21]. Reciprocally, *P. aeruginosa* exoproducts, 4-hydroxy-2-heptylquinoline-*N*-oxide and siderophores, protect *S. aureus* from vancomycin by shifting its metabolism from respiration to fermentation resulting in reduced growth and increased tolerance [22]. Antibiotic inactivation via intracellular antibiotic modification [23], or extracellular hydrolysis [24], can likewise provide protection to sensitive members of synthetic multispecies communities. Alternatively, competitive, or antagonistic interactions, could increase a pathogen’s susceptibility to antibiotics, by altering population dynamics or rate of resistance evolution. For example, inter-species interactions may act synergistically with antibiotics to increase their efficacy [16] by disrupting cross-feeding networks [25], or driving the evolution of susceptibility in otherwise resistant strains [26]. Quantification of the effects of inter-species interactions may help to adapt existing antibiotic treatments to tackle pathogens embedded in multispecies communities.

The association between in vitro antibiotic susceptibility testing and clinical response [15, 27, 28] is lacking in lung infections of patients with cystic fibrosis (CF), partly because the complexity of the CF lung environment is not captured during conventional antibiotic susceptibility testing. A potential reason for this is that CF lung infections are typically polymicrobial, containing multiple different opportunistic pathogens, such as *P. aeruginosa, Haemophilia influenzae, S. aureus* or *Stenotrophomonas maltophilia* that can each contribute towards disease [29]. *P. aeruginosa* is the most common pathogen to chronically infect the CF lung [30] and infections result in prolonged inflammation, bronchiectasis and ultimately respiratory failure [31]. *P. aeruginosa* infections are treated with antipseudomonal antibiotics; however, the impact of coinfecting species upon the outcome of treatment remains poorly understood. Here, we chose to focus on the role of *S. maltophilia*, which is intrinsically multi-drug resistant [32] to most antibiotics used to treat *P. aeruginosa* infections, and estimated to infect between 9 and 18% of CF patients [33]. The impacts of *S. maltophilia* on *P. aeruginosa* infections are currently poorly understood and there is a lack of consensus about its contribution to disease [34], even though coinfections with *P*. *aeruginosa* are rising in prevalence [35–37]. At the Copenhagen Rigshospitalet, *S. maltophilia* has been observed as a marker for lung function decline due to the increased likelihood of secondary *P. aeruginosa* infections [38]. Moreover, *S. maltophilia* more commonly coinfects the CF lung together with *P. aeruginosa* instead of another more comprehensively studied co-occurring species, *S. aureus*. As a result, *S. maltophilia* and *P. aeruginosa* coinfections reflect the real-life clinical situation in the Copenhagen CF Clinic. One explanation for sustained coinfection between these species could be their ability to withstand antipseudomonal drug treatments. Importantly, some resistance mechanisms harboured by *S. maltophilia*, such as two chromosomally encoded β-lactamases [39], have the potential to also provide protection to surrounding sensitive pathogens by detoxifying the environment [20, 40, 41], thereby providing exposure protection.

Here we examine how inter-species interactions between *P. aeruginosa* and the commonly coinfecting CF species S. *maltophilia* (including strains isolated from CF sputum samples) change *P. aeruginosa* susceptibility to carbapenems, imipenem and meropenem. Using model communities in environments mimicking the CF lung in synthetic CF media (SCFM) [42], coupled with mass-spectrometry and mathematical modelling, we quantify and identify drivers of community mediated exposure protection. Though controlled coculture assays, we demonstrate how intrinsic β-lactamase production by *S. maltophilia* can provide antibiotic exposure protection to *P. aeruginosa*. Although antibiotic protection via β-lactamase has been observed previously, here we demonstrate how this effect can directly confound clinically relevant treatments, helping to explain why clinical susceptibility *of P. aeruginosa* is poorly correlated with successful outcomes during the treatment of polymicrobial CF lung infections. Our results show that the ability to survive antibiotic treatment is an emergent community-level property and that the members of CF lung microbiota could either magnify or attenuate the efficacy of antibiotic treatments targeting *P. aeruginosa*.

## Results

### *S. maltophilia* provides imipenem exposure protection to *P. aeruginosa*

**Fig. 1:**
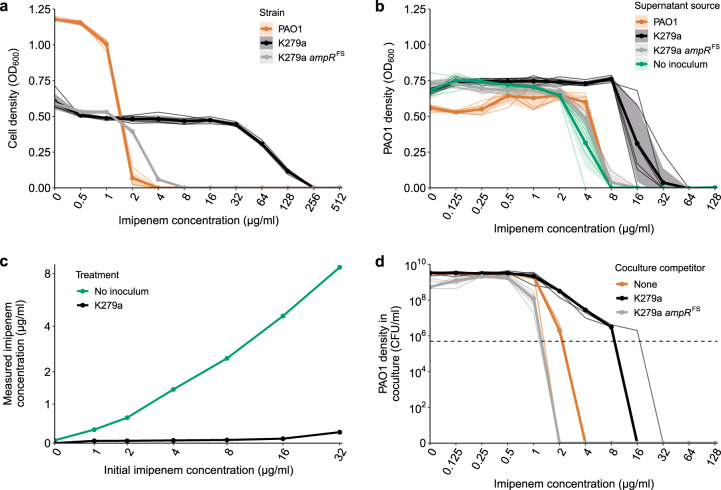
PAO1 exposure protection to imipenem is provided by *S. maltophilia*. **a** Imipenem MIC curves for *P. aeruginosa* PAO1 (orange line), resistant *S. maltophilia* K279a (black line) and susceptible *S. maltophilia* K279a *ampR*^FS^ strains (grey line). **b** Assay to detect the inactivation of imipenem by K279a. The ability of PAO1 to grow in sterile filtered supernatant following 24 h incubation/growth in the presence of imipenem with no inoculum, K279a, K279a *ampR*^FS^ or PAO1. Line colours represent the inoculum of the initial round of growth from which the supernatant was sourced. **c** Measured concentration of imipenem by LCMS in sterile filtered supernatant following 24 h incubation/growth with no inoculum or K279a (*n* = 1). **d** Growth of PAO1 while in coculture with either K279a or K279a *ampR*^FS^ during imipenem treatment, orange line shows PAO1 monoculture control. The horizontal dashed line shows the initial inoculum density of PAO1 (5 × 10^5^ CFU/ml), points above this line show population growth. **a** and **b** bold lines show mean and **d** the median of six biological replicates that are represented by narrow lines of the same colour. **a** and **b** shaded areas show standard deviations (*n* = 6).

We first tested if the *S. maltophilia* K279a strain was able to detoxify the carbapenem imipenem and allow the subsequent growth of sensitive *P. aeruginosa* PAO1. In monoculture, the expression of a chromosomally encoded metallo-L1-β-lactamase, *bla*_L1_, provides K279a with resistance to imipenem (MIC of 256 µg/ml, Fig. 1a), whereas PAO1 is sensitive to imipenem treatment (MIC 2 µg/ml, Fig. 1a). To determine if intrinsic β-lactamase production by *S. maltophilia* can sufficiently detoxify imipenem in the SCFM growth media, we measured PAO1 growth in the supernatant of *S. maltophilia* cultured in the presence of imipenem for 24 h. As a negative control, the susceptible *S. maltophilia* strain K279a *ampR*^FS^ was used, which contains a frameshift in *ampR*, the positive regulator of *bla*_L1_ rendering it unable to express *bla*_L1_ [39]. Culturing wild-type K279a in up to 32 µg/ml imipenem permitted the subsequent growth of PAO1, providing significantly higher levels of protection than the K279a *ampR*^FS^ susceptible strain or the no inoculum negative control (Fig. 1b, post hoc Tukey tests: K279a:K279a *ampR*^FS^ *p* < 0.0001, K279a:No Inoculum *p* < 0.0001). Even though the MIC of PAO1 increased in the supernatant of the sensitive K279a *ampR*^FS^ mutant, the increase was not significantly different from growth in the supernatant of PAO1 or a no inoculum control (Fig. 1b, ANOVA: *F*_3,20_ = 93.12, *p* < 0.001, post hoc Tukey tests: PAO1:No Inoculum *p* = 0.858, K279a *ampR*^FS^:No Inoculum *p* = 0.386). As a result, this difference in MIC was likely due to the natural breakdown of imipenem during the incubation period. These results suggest that K279a was able to detoxify the environment by inactivating imipenem via the expression of L1-β-lactamase and thus allowing the survival of PAO1 at otherwise lethal concentrations of imipenem.

To confirm the breakdown of antibiotic, changes in concentrations of imipenem were measured in monoculture of resistant K279a strains using LCMS after 24 h of growth (Fig. 1c). In the absence of K279a, the concentration of imipenem decreased naturally to approximately one quater of the initial concentration (Fig. 1c), reflecting the increase in survival of PAO1 observed in the supernatant protection assays without K279a inoculum. In contrast, the presence of K279a resulted in a 97.9% mean reduction in imipenem concentration across all concentrations tested (Fig. 1c). This confirms that K279a is highly effective at hydrolysing imipenem, permitting the growth of PAO1 once the environment has been detoxified.

We next tested whether the inactivation of imipenem by *S. maltophilia* could provide exposure protection to *P. aeruginosa* in cocultures. Key benefits of this approach are that cocultures consider both the rate of antibiotic inactivation by the resistant member of the community and the rate of killing of the sensitive member of the community by the antibiotic. Moreover, direct and indirect competition between the two species may limit the potential benefit that the sensitive species can gain from the resistant protector, which would not be observed in supernatant assays. Coculture MIC assays were conducted replicating the conventional broth microdilution MIC protocol, with the addition of a *S. maltophilia* competitor, which was added at a ratio of 10:1 to PAO1 cells (~5 × 10^6^ CFU/ml, K279a or K279a *ampR*^FS^ inoculated with 5 × 10^5^ PAO1). When in coculture with K279a, PAO1 was able to survive at increased concentrations of imipenem, up to 16 µg/ml (Fig. 1d), which is greater than the EUCAST clinical breakpoint for resistance (>4 µg/ml). Notably, although the variance of PAO1 MIC during coculture with K279a was greater than in monoculture, PAO1 was able to maintain a growth at imipenem concentrations that would have otherwise been lethal across all replicates. Growth of *P. aeruginosa* above lethal concentrations was not due to the evolution of resistance as PAO1 isolated from cocultures remained sensitive to 4 µg/m imipenem in monoculture. Moreover, supernatant of *S. maltophilia* cultures grown in the absence of imipenem (Supplementary Figs. S1 and S2), or heat inactivated *S. maltophilia* (Supplementary Fig. S3), did not provide any additional protection to PAO1, showing that actively growing cocultures were required for exposure protection. In contrast, coculture with the β-lactamase-deficient mutant K279a *ampR*^FS^ increased the sensitivity of PAO1 to imipenem compared to PAO1 in monoculture potentially due to competition (Fig. 1d). Together, these results show that PAO1 can benefit from the intrinsic β-lactam resistance encoded by *S. maltophilia* K279a in cocultures, permitting a subpopulation of sensitive *P. aeruginosa* to survive above its monoculture MIC.

As exposure protection to imipenem relies on inactivating the antibiotic, resistance mechanisms that do not alter the environmental concentration of the antibiotic would not be expected to provide protection to susceptible species. To test this hypothesis, we examined the extent of protection provided by *S. aureus* to colistin (Supplementary Fig. S4). The Gram-positive bacterium *S. aureus* is intrinsically resistant to colistin due to the lack of an outer membrane or LPS (Supplementary Fig. S4a) and therefore, unlike the intrinsic carbapenem resistance of *S. maltophilia*, the concentration of colistin in the growth environment is unaltered by the presence of *S. aureus*. As expected, coculturing PAO1 with *S. aureus* did not provide protection from colistin. Rather, the presence of *S. aureus* made PAO1 more susceptible to colistin, decreasing its MIC by half from 4 to 2 µg/ml (Supplementary Fig. S4b), a similar effect to the presence of sensitive K279a *ampR*^FS^ during coculture in the presence of imipenem. Thus, a reduction in the environmental concentration of antibiotic by an intrisically resistant coinfecting species is required to provide exposure protection to sensitive *P. aeruginosa*.

### Exposure protection to imipenem is density dependent

**Fig. 2:**
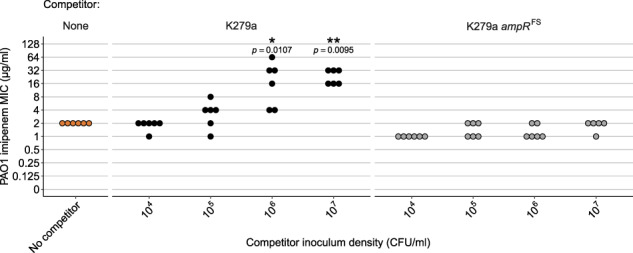
PAO1 exposure protection is dependent on the initial density of resistant K279a. The MIC of PAO1 for imipenem plotted against the initial density of resistant K279a or susceptible K279a *ampR*^FS^ strains. MIC was defined as the concentration of imipenem required to reduce PAO1 growth to below 5% of that in the absence of antibiotic 24 h post inoculation, calculated from broth microdilution cocultures (PAO1 density measured by relative fluorescence; see Supplementary Fig. S5). Points show six independent biological replicates for each condition (five replicates for K279a *ampR*^FS^ at 10^7^ CFU/ml). Stars show a significant difference from the No-Competitor control (*p < 0.05, **p < 0.001), Wilcoxon Rank Sum with Holm–Bonferroni correction was used for multiple comparisons.

MIC values are often strongly dependent upon the initial cell density of the culture, with high populations densities elevating measured MIC values, known as the inoculum effect [4]. The inoculum effect is particularly expected with β-lactams where resistance via hydrolytic inactivation benefits the whole population [43], but it has also been observed with other classes of antibiotics [6]. We hence expected that increasing *S. maltophilia* densities should provide greater protection to *P. aeruginosa*. To test this hypothesis, we manipulated the initial inoculum density of resistant K279a strain covering four orders of magnitude (~10^4^ to ~10^7^ CFU/ml) while maintaining the initial inoculum density of PAO1 the same at 5 × 10^5^ CFU/ml. A low K279a inoculum density of 10^4^ CFU/ml did not provide any protection to PAO1, with no significant difference in MIC between the no-competitor control and coculture treatments (Wilcoxon Rank Sum, Holm–Bonferroni correction, *W* = 15, *p* = 0.878, Fig. 2). Increasing the K279a inoculum density significantly increased the MIC of PAO1 (*F*_1,42_ = 75.11, *p* < 0.001, adjusted *R*^2^ = 0.665), with a maximum PAO1 MIC of 32 µg/ml when in coculture that was inoculated with 10^7^ CFU/ml K279a (Fig. 2 and Supplementary Fig. S5). In contrast, no increase in PAO1 MIC was observed at any of the susceptible K279a *ampR*^FS^ strain inoculum densities (*F*_1,43_ = 0.557, *p* = 0.471, *R*^2^ = 0.021). Together, these results show that the protection was dependent upon the expression of β-lactamase by *S. maltophilia*, and that higher levels of imipenem protection were obtained with increasing *S. maltophilia* density.

### Exposure protection depends on the antimicrobial dose-response and rate of antimicrobial inactivation

**Fig. 3:**
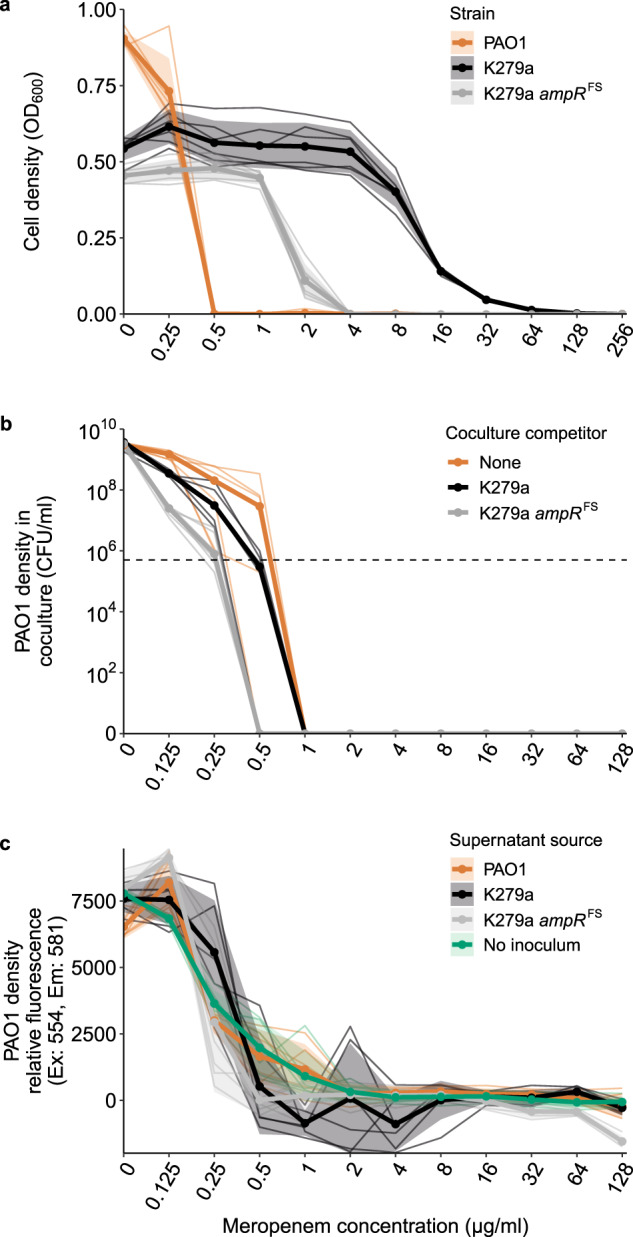
*S. maltophilia* provides no protection to meropenem. **a** Meropenem MIC curves for PAO1*, S. maltophilia* K279a and *S. maltophilia* K279a ampR^FS^. **b** Growth of PAO1 while in coculture with either K279a or K279a ampR^FS^ during meropenem treatment, orange line shows PAO1 monoculture control. **c** Assay to detect the inactivation of meropenem by K279a. The ability of PAO1 to grow in sterile filtered supernatant following 24 h incubation/growth in the presence of meropenem with no inoculum, K279a, K279a ampR^FS^ or PAO1. Line colours represent the inoculum of the initial round of growth from which the supernatant was sourced. **a** and **c** bold lines show mean and **b** the median of six biological replicates that are represented by narrow lines of the same colour. **a** and **c** the shaded areas show standard deviations (*n* = 6). **b** the horizontal dashed line shows the initial inoculum density of PAO1 (5 × 10^5^ CFU/ml).

To determine if protection via *S. maltophilia* L1-β-lactamase was general to other carbapenems, we tested the ability of K279a to provide protection to another commonly used antipseudomonal carbapenem, meropenem. As with imipenem, *S. maltophilia* is intrinsically resistant to meropenem due to the induced expression of L1 β-lactamase (MIC of 64 µg/ml, Fig. 3a), while the *bla*_L1_-deficient mutant K279a *ampR*^FS^ is more sensitive (MIC of 4 µg/ml; Fig. 3a) and PAO1 the most sensitive to meropenem (MIC of 0.5 µg/ml, Fig. 3a). Coculturing PAO1 with K279a did not affect the MIC of PAO1 (Fig. 3b). However, the expression of *bla*_L1_ by K279a compensated for the negative, competition-mediated effects observed during coculturing with the K279a *ampR*^FS^ mutant, which decreased the ability of PAO1 to grow in the presence of meropenem (Fig. 3b). Similarly, supernatant inactivation assays show that K279a was not able to sufficiently inactivate meropenem over 24 h to significantly increase the MIC of PAO1 above 0.5 µg/ml (ANOVA of AUC: *F*_3,20_ = 2.305, *p* = 0.108, post hoc Tukey tests: K279a:No inoculum *p* = 0.606, Fig. 3c). Together, these results indicate that exposure protection critically depends on the inhibitory activity of antibiotics, even when they can be detoxified through the same mechanism.

To develop a quantitative understanding of whether the rate of antibiotic killing and the shape of the dose-response curve of an antibiotic are sufficient to explain the difference in exposure protection dynamics observed between imipenem and meropenem, we developed a simple mathematical ordinary differential equation (ODE) model. The model describes the growth of two species, an antibiotic sensitive species (*S*) and an antibiotic-resistant species (*R*) that inactivates the antibiotic (*A*). We adapted an existing ODE model of Lotka–Volterra competition that captures the ecological dynamics of a two species community [44, 45] and augmented the model with explicit inclusion of pharmacodynamic functions to describe antibiotic action on the sensitive species—the slope of which is described by a Hill-coefficient (*k*) [46]—and inactivation of antibiotics by a resistant species following Michaelis–Menten kinetics. For a full description of the model see Supplementary information, Supplementary Figs. S10–S14, and Supplementary Eqs. 1–3.

**Fig. 4:**
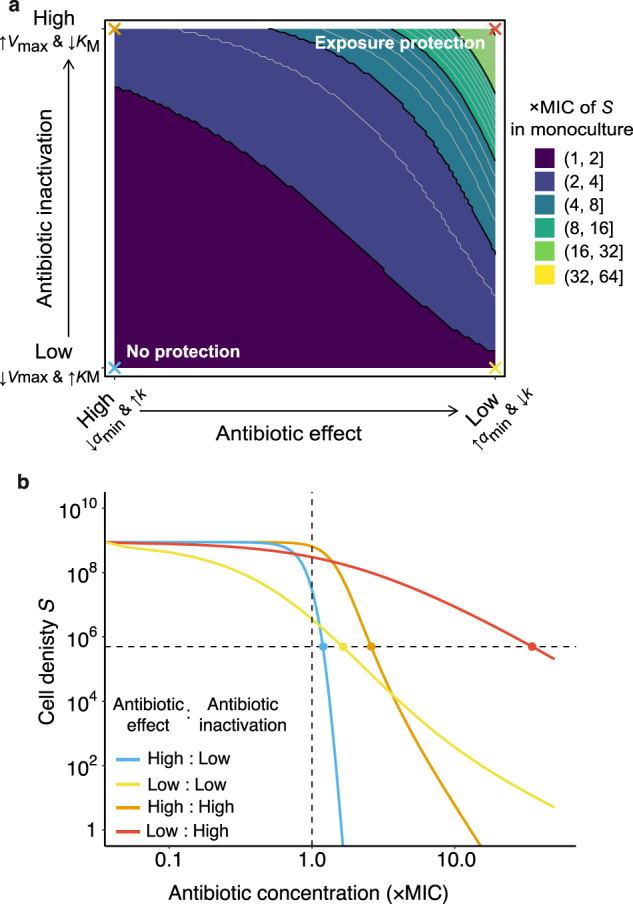
Modelling the exposure protection as a combined function of antibiotic effect and inactivation rate. **a** The *x*-axis plots increasing α_min_ and decreasing *k* that produce a shallower dose-response curve and reduced antibiotic killing rate respectively (lower antibiotic effect, Supplementary Fig. S10) and the *y*-axis plots increasing *V*_max_ and decreasing *K*_M_ that increases the rate of antibiotic inactivation (high antibiotic inactivation). Altering α_min_ and *k* parameters fivefold 1 to 5, and −1.5 to −7.5, respectively, and *V*_max_ and *K*_M_ parameters tenfold 1 × 10^−7^ to 1 × 10^−6^, and 10 to 100, respectively (Supplementary Fig. S14). Shading represents the level of protection provided to the sensitive species *S* when in coculture as the times increase in MIC of *S* in monoculture. Initial density *R* = 5 × 10^6^, initial density *S* = 5 × 10^5^, **b** Combined effect of high effect antibiotics and low inactivation rates for selected values indicated by crosses in panel **a**. Vertical dashed line shows MIC of sensitive population in monoculture and horizontal dashed line shows initial inoculum size of the sensitive population. The MIC is the point at which the cell density of *S* is reduced below the inoculum density, i.e., the net growth rate is zero. Antibiotic high effect: α_min_ = −7.5, *k* = 5, low effect: α_min_ = −1.5, *k* = 1, antibiotic inactivation high rate: *V*_max_ = 1 × 10^−6^, *K*_M_ = 10, low rate: *V*_max_ = 1 × 10^−7^, *K*_M_ = 100, for other parameters see Supplementary Table S2.

Our main finding was that the extent of exposure protection provided to the sensitive species increases with higher rates of antibiotic inactivation and with decreasing antibiotic effect, i.e., shallower dose-response curve and reduced killing rate (Fig. 4a). Steep dose-response curves and high killing rates, which have previously been shown to be desirable properties of antimicrobials to reduce the rate of resistance evolution [47], also reduce the level of exposure protection provided by antibiotic inactivation (Fig. 4a). Higher effect antibiotics kill a larger proportion of the sensitive population before the environment is fully detoxified by the resistant species, resulting in lower levels of exposure protection (Fig. 4b and Supplementary Fig. S11). Likewise, if the antibiotic inactivation rate is not sufficiently high, the environment remains toxic to the sensitive species and the presence of detoxifying species does not significantly increase the MIC of the sensitive strain (Fig. 4b).

These results show that shallow dose-response curves, low killing rates and rapid antibiotic inactivation can result in exposure protection being provided to sensitive species within a community. *S. maltophilia* is able to provide exposure protection to imipenem as the antibiotic is rapidly inactivated (Fig. 1) and has shallow dose-response curve when compared to meropenem [48], resulting in sub-inhibitory concentrations of antibiotic having a lower effect on *P. aeruginosa* growth (Fig. 1a). In contrast, meropenem has a steeper dose-response curve in *P. aeruginosa* (Supplementary Fig. S6); this coupled with a reduced rate of detoxification observed in the supernatant protection assays (Fig. 3c) results in no exposure protection being provided by *S. maltophilia* despite the same mechanism of resistance. The extent of exposure protection thus depends both on the pharmacokinetics, the breakdown of the antibiotic that is influenced by the community composition, and the pharmacodynamics, the response of the sensitive species to antibiotic treatment.

### Clinical *S. maltophilia* isolates from CF sputum provide exposure protection

**Fig. 5:**
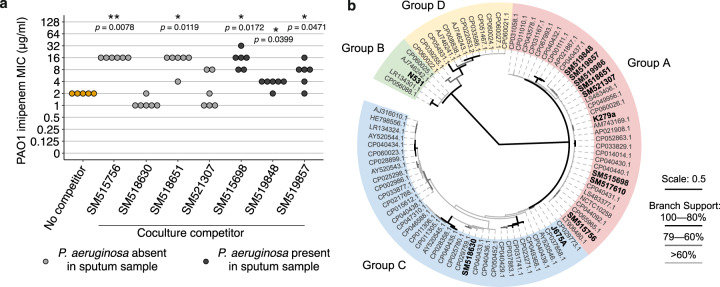
Exposure protection provided by clinical *S. maltophilia* CF isolates is lineage specific and varies between patients. **a** The MIC of PAO1 to imipenem when cocultured with clinical *S. maltophilia* isolates originating from different CF patients. MIC was defined as the concentration of imipenem required to reduce growth to below 5% of that in the absence of antibiotic 24 h post inoculation, calculated from broth microdilution cocultures, PAO1 density measured by relative fluorescence (see Supplementary Fig. S7 and Methods). Points are coloured by whether *P. aeruginosa* was co-isolated along with *the S. maltophilia* isolate from sputum samples, grey no *P. aeruginosa* present, black *P. aeruginosa* co-isolated with *S. maltophilia*. Individual MIC curves for each *S. maltophilia* isolate are presented in Supplementary Fig. S9. Stars show a significant difference from the No-Competitor control and Wilcoxon Rank Sum with Holm–Bonferroni correction was used for multiple comparisons (*p < 0.05, **p < 0.001). **b** An unrooted maximum likelihood phylogeny build using PhyML 3.0 on the *smeT–smeD* intergenic region of 76 publicly available *S. maltophilia* genomes accessible on NCBI together with nine CF *S. maltophilia* isolates from this study labelled in bold. The tree labels are coloured by the phylogenetic groups described by Gould et al. (2006), group-representatives K279a (Group A), N531 (Group B) and J675a (Group C) are labelled in bold. Branch line widths represent percentage bootstrap support, branching for the four major clades are well supported. The scale bar represents 0.5 substitutions per site within the smeT–smeD intergenic region.

To set our results into a clinical context, we explored whether *S. maltophilia* CF isolates can also provide exposure protection to PAO1 against imipenem. We isolated seven *S. maltophilia* isolates that were culturable in SCFM from sputum samples from seven Danish CF patients, three of which were found to co-exist with *P. aeruginosa* (Supplementary Table S1). The level of exposure protection provided by the *S. maltophilia* isolates was strain-specific (Fig. 5a and Supplementary Fig. S7). Five of the seven *S. maltophilia* isolates provided exposure protection to PAO1, increasing the PAO1 MIC in cocultures between two- and eight-fold, up to 16 µg/ml (Fig. 5a). The level of protection provided by these clinical isolates was not associated with their ability to grow in SCFM, level of imipenem resistance in monocultures (Kendall’s Rank Correlation: *τ*_*b*_ = 0.175, *p* = 0.133, Supplementary Figs. S8 and S9), or the co-occurrence with *P. aeruginosa* in the sputum of patients (Wilcoxon Rank Sum: *W* = 239, *p* = 0.555). However, lack of protection could be explained partly by genetic differences between *S. maltophilia* isolates. For example, no genes homologous to *bla*_L1_ were identified within the genome of SM521307, an isolate that provided little protection to PAO1 (Fig. 5a). Furthermore, isolate SM518630, which provided no protection to PAO1, harboured a *bla*_L1_ gene that was genetically distinct from K279a and the other CF clinical isolates, only sharing 87% identity with *bla*_L1_ of K297a, while the remaining strains shared 99% identity (Supplementary Table S1). In addition, SM518630 clustered in Group C based on the *smeT–smeD* intergenic sequence (remaining six isolates again closely resembled K279a in Group A; Fig. 5b). It has previously been shown that *bla*_L1_ activity of strains belonging to *smeT–smeD* Group C is approximately one third of the level observed with isolates from Group A due to low-level constitutive expression of L1 β-lactamase [49]. Together, these findings suggest that while most clinical *S. maltophilia* strains could provide high levels of exposure protection to *P. aeruginosa*, this effect was *S. maltophilia* lineage specific, varying between different CF patients.

## Discussion

Here we studied the efficacy of antibiotic treatment of *P. aeruginosa* during coculture with the pathogen *S. maltophilia* that commonly coinfects the CF lung. We found that coinfecting species can significantly alter the susceptibility of *P. aeruginosa* to antibiotics. During coculture, intrinsically resistant *S. maltophilia* K279a provided density-dependent exposure protection to sensitive *P. aeruginosa*, increasing its MIC to imipenem by up to 16-fold. This level of protection was also typically provided by *S. maltophilia* strains isolated from the CF sputum due to the intrinsic expression of a metallo-L1-β-lactamase that detoxified the environment. Exposure protection was dependent upon both the efficacy of the antibiotic and the rate of antibiotic inactivation by the protective species. In contrast, β-lactam sensitive *S. maltophilia* or intrinsically colistin-resistant *S. aureus* magnified the effect of imipenem and colistin, respectively, likely due to intensified competition. Together, these results suggest that rather than being exclusively a property of an individual strain, antibiotic susceptibility is an emergent property rising from both the phenotype of the pathogen and its interactions with the surrounding community.

The antimicrobial susceptivity tests conducted here provide a more accurate representation of the conditions faced within the CF lung by considering the effect of the most commonly co-occurring pathogens during polymicrobial infection. The results reveal important ecological factors that will contribute to *S. maltophilia*-mediated exposure protection within the site of infection. Firstly, exposure protection by *S. maltophilia* to imipenem was only provided during active coculture, as the expression of *S. maltophilia*-encoded L1 metallo-β-lactamase is typically induced by the presence of β-lactams [39]. Secondly, the protection provided by *S. maltophilia* was dependent upon its density, with only high-density *S. maltophilia* populations providing significant levels of protection to *P. aeruginosa*. Together, these results suggest that for *S. maltophilia* L1 metallo-β-lactamase resistance to provide protection to sensitive *P. aeruginosa* within the CF lung, the two species must be coinfecting the lung concurrently and that *S. maltophilia* must be sufficiently abundant to detoxify the environment. The ecological context of a polymicrobial infection, including knowledge of which species are present at the time of treatment and their abundance, could therefore be important in the choice of effective treatments against pathogens embedded within the community. Co-occurring species were also shown to increases the efficacy of antimicrobial treatment during coculture. *S. aureus*, the second most common species to infect the CF lung in Europe [50], increased the sensitivity of *P. aeruginosa* to colistin, reducing its MIC by half. This reduction in MIC was observed in coculture as well as by the monoculture supernatant of *S. aureus* (Supplementary Fig. S4c), suggesting that *S. aureus* secretions increase the susceptibility of *P. aeruginosa* to colistin. In a similar manner, *P. aeruginosa* exoproducts, LasR endopeptidase and rhamnolipids, can strengthen the antimicrobial effect of vancomycin and tobramycin respectively against *S. aureus* [51]. Such synergistic interactions between co-occurring species and antibiotic usage have the potential to play an important role in the change in community structure over time (i.e., ecological succession) that occurs in the CF lung [52].

The inactivation of carbapenems by *S. maltophilia* was not, however, able to provide protection against meropenem treatment of sensitive *P. aeruginosa* in our assays. Antibiotic stability, inactivation rates and efficacy against *P. aeruginosa* may all have played a role in the differences in protection against imipenem and meropenem. Meropenem has greater stability than imipenem [53], likely resulting in higher levels of exposure for longer periods of time. In addition, meropenem has a relatively higher antimicrobial effect on *P. aeruginosa* than imipenem [48]. These traits of the antibiotic are likely to act together, resulting in a greater reduction in *P. aeruginosa* population prior the protective benefits of environmental detoxification. In addition, *S. maltophilia* resistance to meropenem results from a combination of both hydrolysis via L1 metallo-β-lactamase and efflux via SmeABC and SmeDEF efflux pumps, a mechanism of resistance that has previously been shown to provide no protection to sensitive bacteria during coculture [41]. Our mathematical model highlights the importance of both the inactivation rate of the antibiotic by the resistant species, and the rate of antibiotic killing of the sensitive species. The steeper the dose-response curve of the antibiotic is, the faster the antibiotic inactivation rate required to overcome the initial effect of the antibiotic upon the sensitive population and ultimately provide protection. These results show that antibiotic inactivation via β-lactamase production does not universally provide exposure protection to sensitive species; knowledge of antibiotic pharmacokinetics and pharmacodynamics will therefore be critical in predicting the protective effects provided against different antibiotics.

In addition to carbapenem resistance, *S. maltophilia* also encodes resistance to aminoglycosides via a phosphotransferase [54] and an acyltransferase [55], and both mechanisms of resistance inactivate antibiotics intracellularly. Mixed results have been reported on the ability of intracellular inactivation to provide protection to sensitive community members; intracellular inactivation of chloramphenicol via acetyltransferase has previously been shown to provide cross-species protection [23]. In contrast, aminoglycoside resistance via adenylyltransferase has been shown not to provide protection between resistant and sensitive strains of *P. aeruginosa* [56]. Whether *S. maltophilia* can provide protection against other major classes of antibiotics, such as aminoglycosides, remains to be tested. However, potential protection will still likely depend on the two key parameters described by the model—the rate of inactivation of the antibiotic and rate of antibiotic killing of the sensitive species.

CF clinical *S. maltophilia* isolates provided significant levels of protection against imipenem treatment in most cases, and lack of protection was associated with the absence or low expression of *bla*_L1_. As of 2018, on an average 8.8% of adults with CF across Europe were infected with *S. maltophilia* and the infection rate is as high as 27.1% in Denmark [38, 50]. While coinfections with *S. maltophilia* and *P. aeruginosa* have been reported to be associated with poorer clinical outcomes in CF [57], there is no clear consensus on the clinical impact of *S. maltophilia* [34, 58, 59]. Due to the ambiguity in its contribution to disease state and difficulty in treatment, in the UK *S. maltophilia* treatment is only advised where clinical deterioration is present in the absence of other causes [34]. Our results suggest that *S. maltophilia* coinfections could be partly explained by exposure protection to antibiotics, which may be equally important for *P. aeruginosa* survival as the de novo evolution of chromosomal resistance. Interrupting the interaction between these species will be challenging due to the difficulty of *S. maltophilia* treatment: co-trimoxazole is the only ECAST clinically approved treatment, and L1-beta-lactamase is resistant to all clinically available beta-lactamase inhibitors [60]. As a result, other alternative treatments might need to be developed against *S. maltophilia* such as phage therapy [61].

Although our study attempts to more closely replicate the polymicrobial nature of the CF lung, it has a number of limitations. First, in addition to variation in clinical *S. maltophilia* isolates, *P. aeruginosa* displays a high level of genotypic and phenotypic diversity, particularly during chronic infection [62–65]. Here we used the tractable model strain PAO1, which represents an acute, lung naive *P. aeruginosa* isolate that is highly competitive. To what extent changes in competitiveness during diversification and coevolution alters the dynamics of inter-species exposure protection requires further work. Second, spatial structure is likely to have a large impact on the strength of inter-species interactions for example by privatising protective resistance mechanisms to clonal patches of spatially segregated populations [66, 67]. Alternatively, multispecies biofilms can significantly increase levels of tolerance to antibiotics [68] and thereby allow sensitive species to persist while a resistant species detoxifies the environment. More widely the inherent compartmentalisation, limited mixing and spatial structure of the CF lung may result in separate sites of infection for different species with subsequent mixing only occurring during expectoration [69]. The use of use of mucin and DNA to more closely replicate sputum [70, 71], utilising ex vivo pig lung models to introduce spatial structure [72, 73], or introducing immune responses in the form of in vivo models [74], CFTR mutant cell lines or organoids [75] will provide a more detailed understanding of the inter-species interactions that alter antibiotic efficacy.

Our findings demonstrate that antibiotic susceptibility is an emergent property determined by both the properties of a pathogen and the ecological context in which susceptivity is quantified. While previous research has focused mainly the role of abiotic context, we here show that the presence of other species can change the pathogen’s susceptibility to therapeutic antibiotics. Such interactions will not only alter the efficacy of antibiotic treatment but can also alter the selective pressure imposed by antibiotics, potentially leading to alternative trajectories of antibiotic resistance evolution by changing the strength of antibiotic selection or inducing tolerant cell states [76, 77]. To understand both antibiotic susceptibility and the subsequent evolution of resistance within clinical settings, it is important to consider antibiotic resistance as a community-level trait, which acknowledges the impact of non-pathogenic species in shaping pathogens’ responses to antibiotic treatments.

## Methods

### Strains and media

All experiments with *P. aeruginosa* were conducted using a chromosomally labelled PAO1 isolate. A dTomato and gentamycin resistance cassette was inserted at the neutral attB site using Tn7 homologous recombination as described in Chio and Schweizer [78] using pUC18T-mini-Tn7T-dTomator vector and pTNS2 helper plasmids provided by Prof. Michael Brockhurst. Insertion containing strains were isolated on LB plates containing 30 µg/ml Gentamycin and confirmed by PCR using primers P_Tn7R_ plus P_*glmS*-down_ and P_Tn7L_ + P_*glmS-*up_, as described in Chio and Schweizer [78]. Whole genome sequencing confirmed that no off-target mutations were introduced during homologous recombination and that the dTomato and gentamycin resistance cassette was inserted correctly at the attB site.

*S. maltophilia* strains used in co-included MIC experiments included wild-type *S. maltophilia* K279a with inducible L1 and L2 β-lactamases and β-lactamases deficient *S. maltophilia* K279a *ampR*^FS^ mutant that harbours a 121-bp frameshift inducing deletion within the *ampR* gene [39]. Both strains were provided by Prof. Matthew Avison, University of Bristol. In addition, clinical isolates of nine *S. maltophilia* were isolated from CF sputum samples from nine patients; provided by Prof. Helle Krogh Johansen, Rigshospitalet, Copenhagen; on low salt LB agar plates (NaCl 0.5 g/l) containing 32 µg/ml imipenem, 2.5 µg/ml amphotericin B and 5 µg/ml vancomycin. In addition, one *S. aureus* isolate was isolated on mannitol salt agar from sample 521307. To confirm the species of the isolates 16S rRNA gene was amplified using the 16SA1 (5’-AGAGTTTGATCMTGGCTCAG-3’) and 16SB1 (5’-TACGGYTACCTTGTTACGACTT-3’) primers, purified and Sanger sequenced (GATC, LightRun sequencing). All 16S rRNA sequences of *S. maltophilia* isolates had >99% identity to K279a. In addition, genomic DNA was isolated from 1-ml overnight cultures of each clinical isolate using the Qiagen DNeasy Blood & Tissue extraction kit following the manufacturer’s Gram-negative protocol. Total DNA was sequenced by MicrobesNG using HiSeq (Illumina) and de novo assembly was performed using SPAdes version 3.7 [79] and annotated using Prokka [80] (https://microbesng.com/). All strains were streaked out on to low salt LB agar plates from 25% glycerol stocks, and single colonies were picked for subsequent liquid growth. All liquid overnight cultures were inoculated from individual colonies and growth was conducted at 37 °C and shaken at 180 rpm in 5 ml SCFM, prepared following the protocol outlined in [42] with the addition of thiamine (1 mg/l), nicotinic acid (1.2 mg/l), calcium pantothenate (0.25 mg/l) and biotin (0.005 mg/l) to support the growth of *S. aureus* [81], unless otherwise stated. All antibiotic stocks were prepared on the day of use to avoid degradation of the stock solutions. All growth in 96-well plates was conducted at 37 °C with shaking at 550 rpm with an orbital radius of 3 mm.

### *smeD–smeT* phylogeny construction and *bla*_L1_ comparison

The *smeD–smeT* phylogeny was created using *smeD–smeT* intergenic region from 76 publicly available *S. maltophilia* genomes (GenBank accession number labelled in Fig. 5b) as well as the group-representatives described by Gould et al. (2006) K279a (Group A), N531 (Group B) and J675a (Group C) and nine CF *S. maltophilia* isolates sequenced in this study. Multiple sequence alignment was conducted using MUSCLE alignment algorithm in MEGA version X [82] using default parameters and phylogenetic construction was conducted using PhyML 3.0 maximum likelihood method [83]. The substitution model was automatically selected using ‘Smart Model Selection’ Bayesian Information Criterion [84] and branch support was calculated by bootstrapping 1000 times. The sequence identity of *bla*L1 genes between K279a and the CF isolates was determined by blastn using the de novo assemblies as user-defined databases and K279a *bla*_L1_ as the query sequence on the NCBI Genome Workbench [85]. As no homologous gene to K279a *bla*_L1_ was identifiable in SM521307, reads were mapped to K279a reference (AM743169.1) using BWA-MEM [86] and regions of zero coverage were identified between 2.51 and 2.8 Mb (K279a *bla*_L1_ is located at 2,691,975 bp).

### MIC measurements

All monoculture-MICs were conducted using the broth microdilution method in SCFM with six independent replicates per isolate. Overnight cultures grown in SCFM were inoculated into fresh SCFM containing a two-fold dilution series of antibiotic in 96-well plates to achieve a final volume of 200 µl and a cell density of 5 × 10^5^ CFM/ml, requiring a 1 in 1500 dilution. Cultures were then incubated for 20 h at 37 °C. OD_600_ was measured using a Tecan Infinite Pro 200 microplate reader.

### Antibiotic inactivation assay

To test the ability of *S. maltophilia* to inactivate imipenem during 24 h of growth in SCFM we inoculated two-fold dilution series of imipenem (0–128 µg/ml) with either K279a, K279a *ampR*^FS^ or PAO1 to a density of 5 × 10^5^ CFM/ml in deep-well 96-well plates with a final volume of 800 µl, with six independent replicates per isolate. In addition, a control plate with no inoculum was prepared in parallel. These were incubated for 20 h at 37 °C. Then, 200 µl of the culture was moved into fresh 96-well plates and OD_600_ was measured. The remaining 600 µl was centrifuged at 4500 rpm for 10 min to pellet the bacteria, after which 400 µl of supernatant was removed and passed through 96-well 0.2 µm filter microplates (Agilent filter microplate, 203980-100, Agilent receiver plate 204601-100) by centrifugation at 3000 rpm for 2 min, 190 µl of each sterile filtered supernatant (6× per isolate) was then inoculated with 10 µl of fresh PAO1 overnights diluted 1:75 in SCFM to give a final dilution of 1:1500 and an approximate density of 5 × 10^5^ CFM/ml. The inoculated supernatant plates were then incubated for a further 20 h at 37 °C, after which cell density was measured by OD_600_ using a Tecan Infinite Pro 200 microplate reader.

### LCMS

PAO1 or K279a were inoculated in SCFM containing two-fold dilution series of imipenem from 0 to 32 µg/ml to an initial density of 5 × 10^5^ CFM/ml in a 96-well plate, with two replicates per isolate. Cultures were incubated for 20 h. In parallel, a 0–32 µg/ml dilution series of uninoculated imipenem in SCFM was incubated as above. Following incubation, the cultures were centrifuged at 4500 rpm for 10 min to pellet the bacteria and 150 µl of supernatant was passed through a 96-well 0.2 µm filter microplate. The supernatants from the same treatment were pooled in preparation for LCMS. Samples were analysed on an Acquity IClass LC (Waters, Elstree, UK), which was connected to an Orbitrap Fusion MS (ThermoFisher, Altrincham, UK). Data were acquired in positive ESI ionisation in DDA mode (alternating HCD and CID fragmentations) at a cycle time of 0.4 s and master scans at a resolution of 60 K. The protonated precursor of m/z 300.1 was used to quantify IMI in the samples, using software package Thermo Xcalibur 4.0; the MS2 fragments of IMI, 141.9 and 194.9, were used for identification purposes. Mobile phase A was water with 0.1% acetic acid, B acetonitrile with 0.1% acetic acid. Phase B started at 0% for 0.2 min, was ramped up to 95% until 7 min, stayed isocratic until 8 min, was brought down to 0% at 8.1 min and stayed there until 9 min. Flow rate was 0.5 ml/min. The column was a HSS T3 100×2.1, 1.7 (Waters) at 40 °C; injection volume was 2 μl. Concentrations of imipenem within each sample were calculated using an imipenem standard curve prepared in SCFM.

### Coculture protection assays

To test for changes in susceptibility of PAO1 while in coculture, overnight cultures of the competitor strains were used to inoculate SCFM in rows of a 96-well plates to finial inoculum density of ~1 × 10^5^ CFU/ml, with six independent replicates per competitor strain. Competitors were incubated at 37 °C with for 6 h, resulting in a competitor density of ~5 × 10^6^ CFU/ml. In parallel, a separate control plate with no inoculum was prepared and incubated as above. Following competitor growth, a two-fold dilution series of antibiotics (imipenem, meropenem or colistin) was added to the competitor strains to a final concentration of between 0 and 128 µg/ml. The competitor strain pre-culture plus antibiotic mixes were then immediately inoculated with PAO1 to a final density of 5 × 10^5^ CFU/ml, resulting in ~10:1 ratio of competitor to PAO1. The cocultures were incubated for a further 20 h at 37 °C. PAO1 density was then measured either by plating out the cultures to calculate CFU/ml (Figs. 1d and 3b and Supplementary Figs. S3 and S4b) or estimated by measuring red fluorescence signal (Figs. 2, 3c and 5a). For CFU measurements, cocultures were serial diluted in PBS using a Gilson Platemaster and spotted onto Pseudomonas Selective Agar plates, which were incubated for 10–16 h until colony growth was visible. The threshold for detection was ~2 × 10^5^ CFU/ml. For fluorescence measurements, the RFP signal of the cocultures was measured using a Tecan Infinite Pro 200 plate reader, with an excitation wavelength of 552 nm and an emission wavelength of 581 nm. The gain for each florescent measurement was maintained across all samples and experiments.

To measure the effect of altering the initial density of *S. maltophilia* during coculture, PAO1 susceptibility was tested following the coculture protection assay protocol as described above with PAO1 density measured by fluorescence. However, rather than pre-growing the competitor strain for 6 h, competitor overnight cultures were diluted to the desired density (1 × 10^4^, 1 × 10^5^, 1 × 10^6^ or 1 × 10^7^ CFU/ml) in SCFM prior to the addition of antibiotic and PAO1 inoculation. Initial densities of *S. maltophilia* competitor were confirmed by plating out for single colonies on low salt LB agar plates.

The ability of heat inactivated *S. maltophilia* cells to provide protection to imipenem was conducted following the coculture protection assay protocol as above; however, prior to the addition of antibiotic and PAO1 inoculum, the 6-h *S. maltophilia* pre-growth cultures were incubated at 100 °C for 10 min, and then allowed to cool to room temperature. The control treatment of SCFM with no inoculum was also incubated to 100 °C for 10 min to control for the effect of heating the media. Neat heat inactivated *S. maltophilia* culture was plated out for single colonies on low salt LB agar plates to confirm complete inactivation.

### Supernatant protection assays

Monocultures of *S. maltophilia, S. aureus* or *P. aeruginosa* were grown in 6 ml of SCFM for 20 h in the absence of antibiotic, with six replicates per strain. Saturated monocultures were centrifuged at 4500 rpm for 10 min to pellet the bacteria and the supernatant was removed and passed through a 0.22-µm filter. The sterile supernatant was then used at the growth media for PAO1 microdilution MIC curves as described above for imipenem and colistin, with six independent replicates per supernatant source.

### Statistics

All statistical analysis was conducted in R 3.6.1. Differences in growth under antibiotic treatment were calculated by ANOVA of the integral of the resistance profiles, with subsequent Tukey multiple comparison of means. Kendall’s Rank Correlation was conducted to test for a correlation between the level of protection provided to PAO1 in coculture with clinical *S. maltophilia* isolates and the integral of *S. maltophilia* MIC curves.

Wilcoxon Rank Sum test was used when normality of data could not be assumed to test for differences in the MIC of PAO1 in monoculture vs coculture treatments, using Holm method correction for multiple comparisons. For the analysis of the effect of density on protection, we fitted a linear model where inoculum density and competitor were fixed effects and MIC of PAO1 was the response variable, with log-transformed MIC and inoculum density.

All CFU data are plotted using the median (thick line) with each individual replicate plotted (thin lines), all OD and relative fluorescence data are plotted using the mean (thick line), with each individual replicate plotted (thin lines) and shaded areas show standard deviations.

## Supplementary information

Supplementary Material

## Data Availability

All raw data are available on FigShare (10.6084/m9.figshare.15164295.v1), and all sequence data generated in this study are accessible at the European Nucleotide Archive (accession: PRJEB47042).

## References

[1] Filkins LM, O’Toole GA (2015). Cystic fibrosis lung infections: polymicrobial, complex, and hard to treat. PLoS Pathog.

[2] Paterson IK, Hoyle A, Ochoa G, Baker-Austin C, Taylor NGH (2016). Optimising antibiotic usage to treat bacterial infections. Sci Rep..

[3] Andrews JM (2001). Determination of minimum inhibitory concentrations. J Antimicrob Chemother.

[4] Brook I (1989). Inoculum effect. Rev Infect Dis.

[5] Karslake J, Maltas J, Brumm P, Wood KB (2016). Population density modulates drug inhibition and gives rise to potential bistability of treatment outcomes for bacterial infections. PLOS Comput Biol..

[6] Udekwu KI, Parrish N, Ankomah P, Baquero F, Levin BR (2009). Functional relationship between bacterial cell density and the efficacy of antibiotics. J Antimicrob Chemother..

[7] Sweeney E, Sabnis A, Edwards AM, Harrison F (2020). Effect of host-mimicking medium and biofilm growth on the ability of colistin to kill *Pseudomonas aeruginosa*. Microbiology.

[8] Walters MC, Roe F, Bugnicourt A, Franklin MJ, Stewart PS (2003). Contributions of antibiotic penetration, oxygen limitation, and low metabolic activity to tolerance of *Pseudomonas aeruginosa* biofilms to ciprofloxacin and tobramycin. Antimicrob Agents Chemother..

[9] Nguyen D, Joshi-Datar A, Lepine F, Bauerle E, Olakanmi O, Beer K (2011). Active starvation responses mediate antibiotic tolerance in biofilms and nutrient-limited bacteria. Science..

[10] Høiby N, Bjarnsholt T, Givskov M, Molin S, Ciofu O (2010). Antibiotic resistance of bacterial biofilms. Int J Antimicrob Agents..

[11] Olsen I (2015). Biofilm-specific antibiotic tolerance and resistance. Eur J Clin Microbiol Infect Dis..

[12] Macia MD, Rojo-Molinero E, Oliver A (2014). Antimicrobial susceptibility testing in biofilm-growing bacteria. Clin Microbiol Infect..

[13] Thieme L, Hartung A, Tramm K, Klinger-Strobel M, Jandt KD, Makarewicz O (2019). MBEC versus MBIC: the lack of differentiation between biofilm reducing and inhibitory effects as a current problem in biofilm methodology. Biol Proced Online..

[14] Bottery MJ, Pitchford JW, Friman V-P (2021). Ecology and evolution of antimicrobial resistance in bacterial communities. ISME J.

[15] Smith AL, Fiel SB, Mayer-Hamblett N, Ramsey B, Burns JL (2003). Susceptibility testing of *Pseudomonas aeruginosa* isolates and clinical response to parenteral antibiotic administration: lack of association in cystic fibrosis. Chest..

[16] Radlinski L, Conlon B (2018). Antibiotic efficacy in the complex infection environment. Curr Opin MicrobioL.

[17] Vos MGJ, de, Zagorski M, McNally A, Bollenbach T (2017). Interaction networks, ecological stability, and collective antibiotic tolerance in polymicrobial infections. PNAS.

[18] Adamowicz EM, Flynn J, Hunter RC, Harcombe WR (2018). Cross-feeding modulates antibiotic tolerance in bacterial communities. ISME J.

[19] Aranda-Díaz A, Obadia B, Dodge R, Thomsen T, Hallberg ZF, Güvener ZT (2020). Bacterial interspecies interactions modulate pH-mediated antibiotic tolerance. eLife..

[20] Vega NM, Gore J (2014). Collective antibiotic resistance: mechanisms and implications. Curr Opin Microbiol.

[21] Beaudoin T, Yau YCW, Stapleton PJ, Gong Y, Wang PW, Guttman DS (2017). *Staphylococcus aureus* interaction with *Pseudomonas aeruginosa* biofilm enhances tobramycin resistance. NPJ Biofilms Microbiomes.

[22] Orazi G, O’Toole GA (2017). *Pseudomonas aeruginosa* alters *Staphylococcus aureus* sensitivity to vancomycin in a biofilm model of cystic fibrosis infection. mBio..

[23] Sorg RA, Lin L, Doorn GS, van, Sorg M, Olson J, Nizet V (2016). Collective resistance in microbial communities by intracellular antibiotic deactivation. PLOS Biol.

[24] Perlin MH, Clark DR, McKenzie C, Patel H, Jackson N, Kormanik C (2009). Protection of *Salmonella* by ampicillin-resistant *Escherichia coli* in the presence of otherwise lethal drug concentrations. Proc R Soc B..

[25] Flynn JM, Cameron LC, Wiggen TD, Dunitz JM, Harcombe WR, Hunter RC (2020). Disruption of cross-feeding inhibits pathogen growth in the sputa of patients with cystic fibrosis. mSphere..

[26] Gurney J, Brown SP, Kaltz O, Hochberg ME (2020). Steering phages to combat bacterial pathogens. Trends Microbiol.

[27] Waters VJ, Kidd TJ, Canton R, Ekkelenkamp MB, Johansen HK, LiPuma JJ (2019). Reconciling antimicrobial susceptibility testing and clinical response in antimicrobial treatment of chronic cystic fibrosis lung infections. Clin Infect Dis.

[28] Somayaji R, Parkins MD, Shah A, Martiniano SL, Tunney MM, Kahle JS (2019). Antimicrobial susceptibility testing (AST) and associated clinical outcomes in individuals with cystic fibrosis: a systematic review. J Cyst Fibros..

[29] Raghuvanshi R, Vasco K, Vázquez-Baeza Y, Jiang L, Morton JT, Li D (2020). High-resolution longitudinal dynamics of the cystic fibrosis sputum microbiome and metabolome through antibiotic therapy. mSystems..

[30] Cystic Fibrosis Trust. UK cystic fibrosis registry annual data report 2019. 2020. [online] Available at: https://www.cysticfibrosis.org.uk/sites/default/files/2020-12/2019%20Registry%20Annual%20Data%20report_Sep%202020.pdf [Accessed 5 June 2021].

[31] Nixon GM, Armstrong DS, Carzino R, Carlin JB, Olinsky A, Robertson CF (2001). Clinical outcome after early *Pseudomonas aeruginosa* infection in cystic fibrosis. J Pediatr.

[32] Sánchez MB (2015). Antibiotic resistance in the opportunistic pathogen *Stenotrophomonas maltophilia*. Front Microbiol..

[33] Salsgiver EL, Fink AK, Knapp EA, LiPuma JJ, Olivier KN, Marshall BC (2016). Changing epidemiology of the respiratory bacteriology of patients with cystic fibrosis. Chest.

[34] Cystic Fibrosis Trust. Antibiotic treatment for cystic fibrosis. 2009. [online] Available at: https://www.cysticfibrosis.org.uk/sites/default/files/2020-11/Anitbiotic%20Treatment.pdf [Accessed 7 June 2021].

[35] Denton M, Todd NJ, Littlewood JM (1996). Role of anti-pseudomonal antibiotics in the emergence of *Stenotrophomonas maltophilia* in cystic fibrosis patients. Eur J Clin Microbiol Infect Dis..

[36] Esposito A, Pompilio A, Bettua C, Crocetta V, Giacobazzi E, Fiscarelli E (2017). Evolution of *Stenotrophomonas maltophilia* in cystic fibrosis lung over chronic infection: a genomic and phenotypic population study. Front Microbiol..

[37] Pompilio A, Crocetta V, De Nicola S, Verginelli F, Fiscarelli E, Di Bonaventura G (2015). Cooperative pathogenicity in cystic fibrosis: *Stenotrophomonas maltophilia* modulates *Pseudomonas aeruginosa* virulence in mixed biofilm. Front Microbiol..

[38] Dalbøge CS, Hansen CR, Pressler T, Høiby N, Johansen HK (2011). Chronic pulmonary infection with *Stenotrophomonas maltophilia* and lung function in patients with cystic fibrosis. J Cyst Fibros..

[39] Okazaki A, Avison MB (2008). Induction of L1 and L2 β-lactamase production in *Stenotrophomonas maltophilia* is dependent on an AmpR-type regulator. Antimicrob Agents Chemother.

[40] Yurtsev EA, Chao HX, Datta MS, Artemova T, Gore J (2013). Bacterial cheating drives the population dynamics of cooperative antibiotic resistance plasmids. Mol Syst Biol.

[41] Bottery MJ, Wood AJ, Brockhurst MA (2016). Selective conditions for a multidrug resistance plasmid depend on the sociality of antibiotic resistance. Antimicrob Agents Chemother..

[42] Palmer KL, Aye LM, Whiteley M (2007). Nutritional cues control *Pseudomonas aeruginosa* multicellular behavior in cystic fibrosis sputum. J Bacteriol.

[43] Artemova T, Gerardin Y, Dudley C, Vega NM, Gore J (2015). Isolated cell behavior drives the evolution of antibiotic resistance. Mol Syst Biol..

[44] Harrison E, Wood AJ, Dytham C, Pitchford JW, Truman J, Spiers A (2015). Bacteriophages limit the existence conditions for conjugative plasmids. mBio..

[45] Hall JPJ, Wood AJ, Harrison E, Brockhurst MA (2016). Source–sink plasmid transfer dynamics maintain gene mobility in soil bacterial communities. PNAS..

[46] Regoes RR, Wiuff C, Zappala RM, Garner KN, Baquero F, Levin BR (2004). Pharmacodynamic functions: a multiparameter approach to the design of antibiotic treatment regimens. Antimicrob Agents Chemother..

[47] Yu G, Baeder DY, Regoes RR, Rolff J (2018). Predicting drug resistance evolution: insights from antimicrobial peptides and antibiotics. Proc R Soc B..

[48] Zhanel GG, Simor AE, Vercaigne L, Mandell L (1998). Imipenem and meropenem: comparison of in vitro activity, pharmacokinetics, clinical trials and adverse effects. Can J Infect Dis..

[49] Gould VC, Okazaki A, Avison MB (2006). β-Lactam resistance and β-lactamase expression in clinical *Stenotrophomonas maltophilia* isolates having defined phylogenetic relationships. J Antimicrob Chemother.

[50] European Cystic Fibrosis Society Patient Registry. ECFS patient registry annual data report 2018. 2020. [online] Available at: https://www.ecfs.eu/sites/default/files/general-content-files/working-groups/ecfs-patient-registry/ECFSPR_Report_2018_v1.4.pdf [Accessed 7 June 2021].

[51] Radlinski L, Rowe SE, Kartchner LB, Maile R, Cairns BA, Vitko NP (2017). *Pseudomonas aeruginosa* exoproducts determine antibiotic efficacy against *Staphylococcus aureus*. PLoS Biol.

[52] Harrison FY (2007). Microbial ecology of the cystic fibrosis lung. Microbiology.

[53] Keel RA, Sutherland CA, Crandon JL, Nicolau DP (2011). Stability of doripenem, imipenem and meropenem at elevated room temperatures. Int J Antimicrob Agents.

[54] Okazaki A, Avison MB (2007). Aph(3′)-IIc, an Aminoglycoside resistance determinant from *Stenotrophomonas maltophilia*. Antimicrob Agents Chemother..

[55] Li X-Z, Zhang L, McKay GA, Poole K (2003). Role of the acetyltransferase AAC(6′)-Iz modifying enzyme in aminoglycoside resistance in *Stenotrophomonas maltophilia*. J Antimicrob Chemother.

[56] Frost I, Smith WPJ, Mitri S, Millan AS, Davit Y, Osborne JM (2018). Cooperation, competition and antibiotic resistance in bacterial colonies. ISME J..

[57] Yin C, Yang W, Meng J, Lv Y, Wang J, Huang B (2017). Co-infection of *Pseudomonas aeruginosa* and *Stenotrophomonas maltophilia* in hospitalised pneumonia patients has a synergic and significant impact on clinical outcomes. Eur J Clin Microbiol Infect Dis..

[58] Waters V, Yau Y, Prasad S, Lu A, Atenafu E, Crandall I (2011). *Stenotrophomonas maltophilia* in cystic fibrosis: serologic response and effect on lung disease. Am J Respir Crit Care Med..

[59] Goss CH, Mayer-Hamblett N, Aitken ML, Rubenfeld GD, Ramsey BW (2004). Association between *Stenotrophomonas maltophilia* and lung function in cystic fibrosis. Thorax.

[60] Mojica MF, Ouellette CP, Leber A, Becknell MB, Ardura MI, Perez F (2016). Successful treatment of bloodstream infection due to metallo-β-lactamase-producing *Stenotrophomonas maltophilia* in a renal transplant patient. Antimicrob Agents Chemother..

[61] McCutcheon JG, Dennis JJ (2021). The potential of phage therapy against the emerging opportunistic pathogen *Stenotrophomonas maltophilia*. Viruses.

[62] Rossi E, La Rosa R, Bartell JA, Marvig RL, Haagensen JAJ, Sommer LM (2021). *Pseudomonas aeruginosa* adaptation and evolution in patients with cystic fibrosis. Nat Rev Microbiol..

[63] Davies EV, James CE, Brockhurst MA, Winstanley C (2017). Evolutionary diversification of *Pseudomonas aeruginosa* in an artificial sputum model. BMC Microbiol.

[64] Bara JJ, Matson Z, Remold SK (2018). Life in the cystic fibrosis upper respiratory tract influences competitive ability of the opportunistic pathogen *Pseudomonas aeruginosa*. R Soc Open Sci.

[65] Bartell JA, Sommer LM, Haagensen JAJ, Loch A, Espinosa R, Molin S (2019). Evolutionary highways to persistent bacterial infection. Nat Commun..

[66] Estrela S, Brown SP (2018). Community interactions and spatial structure shape selection on antibiotic resistant lineages. PLOS Comput Biol..

[67] McNally L, Bernardy E, Thomas J, Kalziqi A, Pentz J, Brown SP (2017). Killing by Type VI secretion drives genetic phase separation and correlates with increased cooperation. Nat Commun..

[68] Burmølle M, Webb JS, Rao D, Hansen LH, Sørensen SJ, Kjelleberg S (2006). Enhanced biofilm formation and increased resistance to antimicrobial agents and bacterial invasion are caused by synergistic interactions in multispecies biofilms. Appl Environ Microbiol..

[69] Willner D, Haynes MR, Furlan M, Schmieder R, Lim YW, Rainey PB (2012). Spatial distribution of microbial communities in the cystic fibrosis lung. ISME J.

[70] Turner KH, Wessel AK, Palmer GC, Murray JL, Whiteley M (2015). Essential genome of *Pseudomonas aeruginosa* in cystic fibrosis sputum. PNAS.

[71] Kirchner S, Fothergill JL, Wright EA, James CE, Mowat E, Winstanley C (2012). Use of artificial sputum medium to test antibiotic efficacy against *Pseudomonas aeruginosa* in conditions more relevant to the cystic fibrosis lung. J Vis Exp..

[72] Harrison F, Diggle SP (2016). An ex vivo lung model to study bronchioles infected with *Pseudomonas aeruginosa* biofilms. Microbiology.

[73] Harrington NE, Sweeney E, Harrison F (2020). Building a better biofilm – formation of in vivo-like biofilm structures by *Pseudomonas aeruginosa* in a porcine model of cystic fibrosis lung infection. Biofilm.

[74] Bricio-Moreno L, Sheridan VH, Goodhead I, Armstrong S, Wong JKL, Waters EM (2018). Evolutionary trade-offs associated with loss of PmrB function in host-adapted *Pseudomonas aeruginosa*. Nat Commun..

[75] Castellani S, Di Gioia S, di Toma L, Conese M (2018). Human cellular models for the investigation of lung inflammation and mucus production in cystic fibrosis. Anal Cell Pathol..

[76] Levin-Reisman I, Ronin I, Gefen O, Braniss I, Shoresh N, Balaban NQ (2017). Antibiotic tolerance facilitates the evolution of resistance. Science..

[77] Wistrand-Yuen E, Knopp M, Hjort K, Koskiniemi S, Berg OG, Andersson DI (2018). Evolution of high-level resistance during low-level antibiotic exposure. Nat Commun..

[78] Choi K-H, Schweizer HP (2006). mini-Tn*7* insertion in bacteria with single *att*Tn*7* sites: example *Pseudomonas aeruginosa*. Nat Protoc..

[79] Bankevich A, Nurk S, Antipov D, Gurevich AA, Dvorkin M, Kulikov AS (2012). SPAdes: a new genome assembly algorithm and Its applications to single-cell Sequencing. J Comput Biol..

[80] Seemann T (2014). Prokka: rapid prokaryotic genome annotation. Bioinformatics..

[81] Onoue Y, Mori M (1997). Amino acid requirements for the growth and enterotoxin production by *Staphylococcus aureus* in chemically defined media. Int J Food Microbiol..

[82] Kumar S, Stecher G, Li M, Knyaz C, Tamura K (2018). MEGA X: molecular evolutionary genetics analysis across computing platforms. Mol Biol Evol.

[83] Guindon S, Dufayard J-F, Lefort V, Anisimova M, Hordijk W, Gascuel O (2010). New algorithms and methods to estimate maximum-likelihood phylogenies: assessing the performance of PhyML 3.0. Syst Biol.

[84] Lefort V, Longueville J-E, Gascuel O (2017). SMS: smart model selection in PhyML. Mol Biol Evol.

[85] Kuznetsov A, Bollin CJ (2021). NCBI Genome Workbench: desktop software for comparative genomics, visualization, and GenBank data submission. Methods Mol Biol.

[86] Li H, Durbin R (2009). Fast and accurate short read alignment with Burrows-Wheeler transform. Bioinformatics..

