# Supplementary material for: Inter-species interactions alter antibiotic efficacy in bacterial communities
Source: ISME J. 2021 Oct 9;16(3):812–21. doi: 10.1038/s41396-021-01130-6 (PMC8857223; doi:10.1038/s41396-021-01130-6)
Supplement: Supplementary file 1 — Supplementary Material [file 41396_2021_1130_MOESM1_ESM.pdf]

Inter-species interactions alter antibiotic efficacy in polymicrobial communities

Michael J. Bottery[1,2], Jessica L. Matthews[2], A. Jamie Wood[2,3], Helle Krogh Johansen[4,5,6], Jon W. Pitchford[2,3] & Ville-Petri Friman[2]

[1]Division of Infection, Immunity and Respiratory Medicine, School of Biological Sciences, University of Manchester, Manchester, M13 9PL, UK.
[2]Department of Biology, University of York, Wentworth Way, York, YO10 5DD, UK.
[3]Department of Mathematics, University of York, Heslington, York, YO10 5DD, UK.
[4]The Novo Nordisk Foundation Center for Biosustainability, Technical University of Denmark, Lyngby, Denmark
[5]Department of Clinical Microbiology 9301, Rigshospitalet, Copenhagen, Denmark
[6]Department of Clinical Medicine, Faculty of Health and Medical Sciences, University of Copenhagen, Copenhagen, Denmark

Supplementary Information

Contents

1 – Experimental Supplementary Figures…… 2
Fig. S1-S9
Table S1
2 – ODE Modelling Supplementary Information…… 9
Equation S1-S3
Fig. S10-S14
Table S2
3 – SI References…… 17

1 – Experimental Supplementary Figures

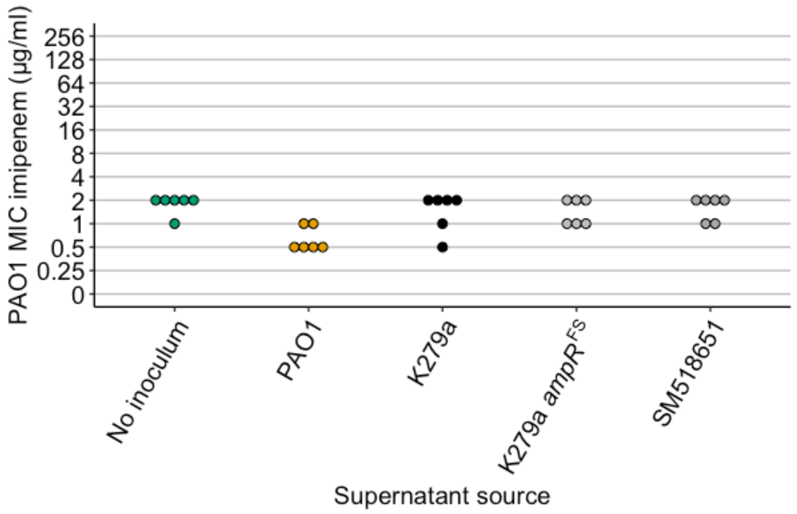

Fig. S1 Supernatant from *S. maltophilia* cultures alone does not provide protection to **i**mipenem. Monocultures of PAO1, K279a K279a *ampR*^{FS} or the *S. maltophilia* CF clinical isolate SM518651 were grown in monoculture in the absence of antibiotic. Imipenem microdilution MIC measurements for PAO1 where then conducted in the sterile filtered supernatant of these cultures. The MIC was defined as concentration required to reduce grow to below 5% of that in the absence of growth in the absence of antibiotic (see Fig. S2). Points show 6 independent biological replicates for each condition.

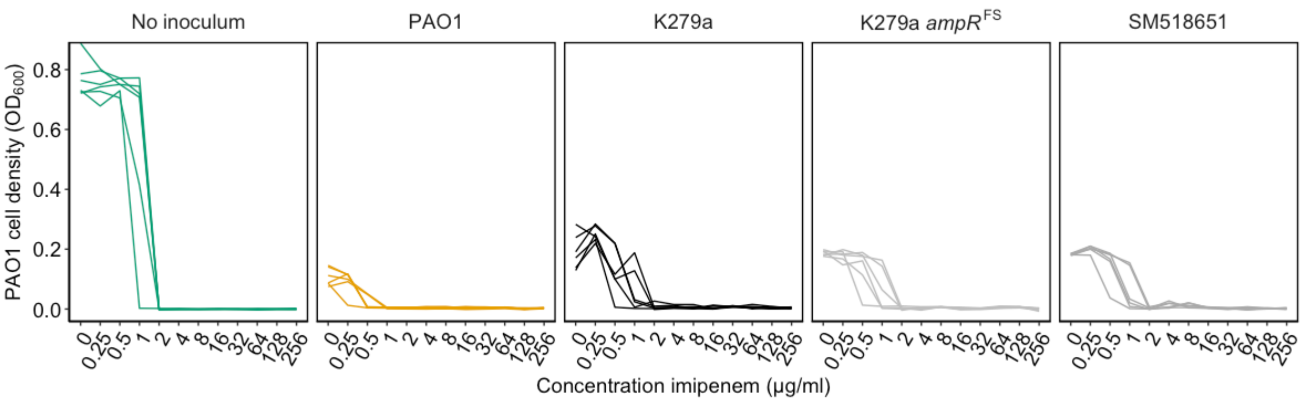

Fig. S2 Growth data (OD_{600}) used to calculate the PAO1 MICs presented in Fig. S1. The columns are faceted by the source of supernatant that the MIC tests were conducted in. Each of the 6 independent replicates for each condition are plotted as separate lines.

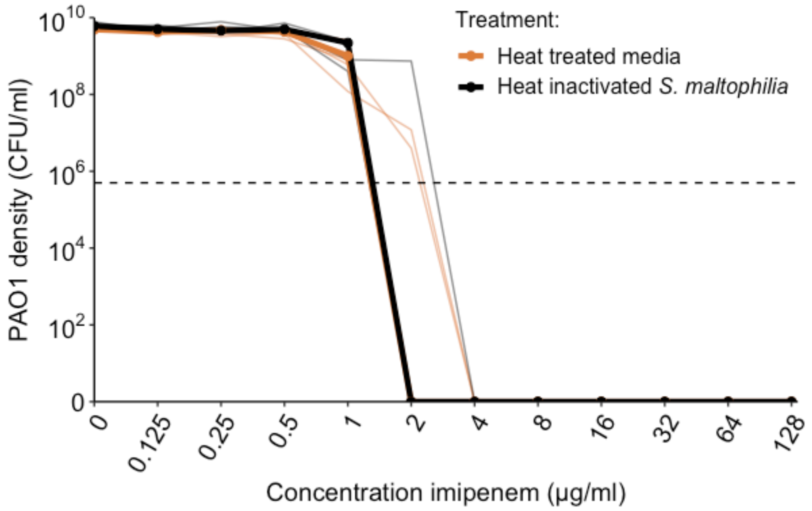

Fig. S3 Heat inactivated cells provide no protection to *P. aeruginosa*. Growth of PAO1 with the addition of $5{\times}10^6$ CFU/ml heat inactivated K279a cells at a ratio of 10:1 to PAO1. The horizontal dashed line shows the initial inoculum density of PAO1 ($5{\times}10^5$ CFU/ml). The control (orange line) shows heat treated media in the absence of *S. maltophilia*. Bold lines show median of 6 biological replicates which are represented by narrow lines of the same colour.

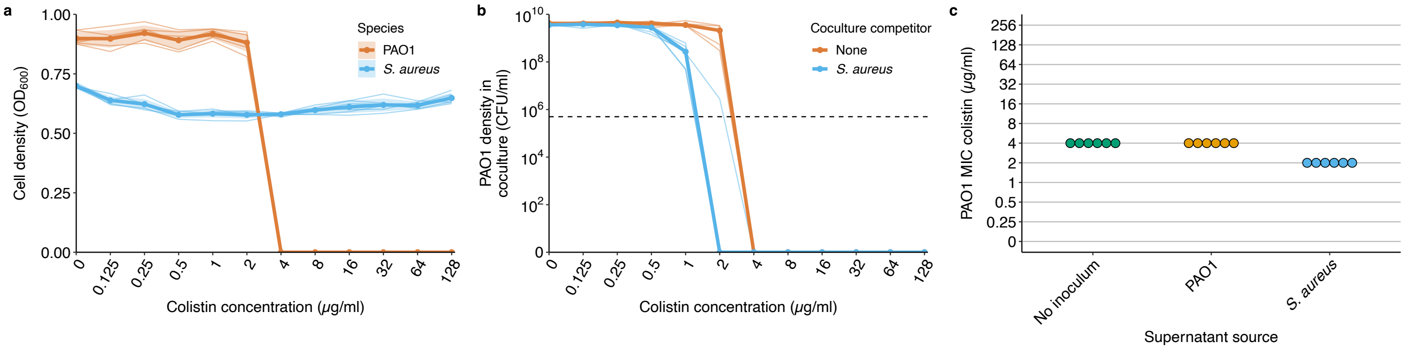

Fig. S4 a Monoculture colistin MIC of PAO1 and CF isolated *S. aureus*. Bold lines show the mean of 6 biological replicates which are represented by narrow lines of the same colour. Shaded areas show standard deviations (n=6). **b** Growth of PAO1 while in co-culture with *S. aureus* during colistin treatment, orange line shows PAO1 monoculture control. Bold lines show the median of 6 biological replicates which are represented by narrow lines of the same colour. The horizontal dashed line shows the initial inoculum density of PAO1 ($5{\times}10^5$ CFU/ml). **c** Colistin MIC measurements of PAO1 conducted in the sterile filtered supernatants of PAO1 or *S. aureus* monocultures. Point colours represent the source of the supernatant and show 6 independent biological replicates per treatment.

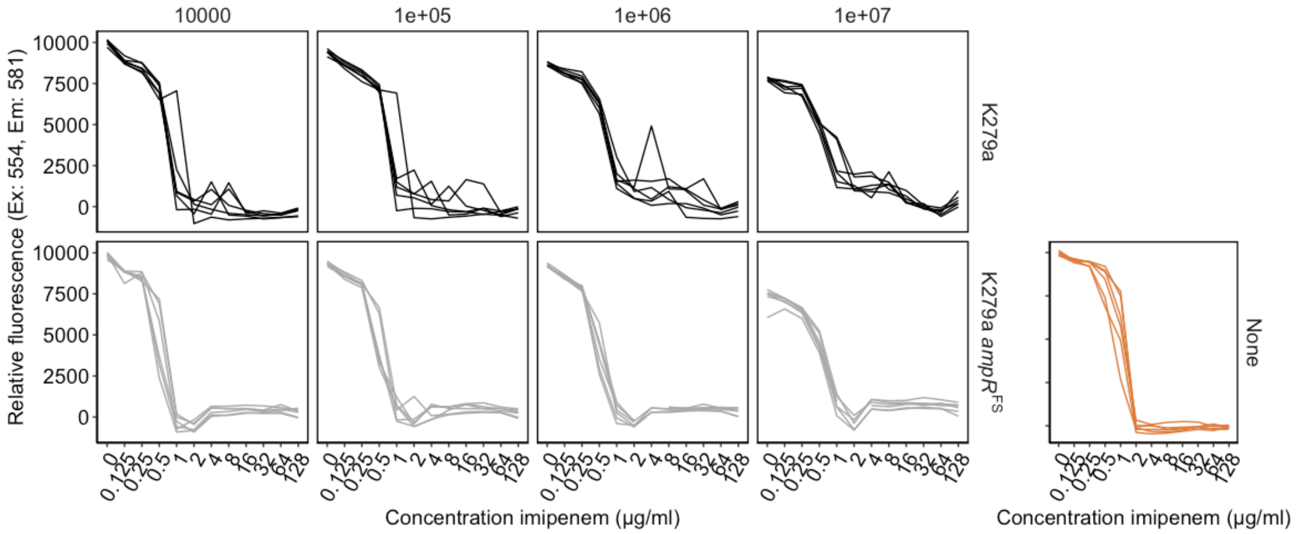

Fig. S5 Growth data used to calculate MICs presented in Fig 2 of the main text. Growth of PAO1 chromosomally labelled with dTomato was detected by red fluorescence. K279a produce no detectable auto-fluorescence at Ex 554, Em 581. The columns are faceted by the initial density of the *S. maltophilia* inoculum density (10^4-10^7) and rows by the *S. maltophilia* strain (K279a or K279a ampR^{FS}). The right plot shows PAO1 fluorescence in the absence of a competitor. Each of the 6 independent replicates are plotted as separate lines (5 replicates for K279a ampR^{FS} 10^7).

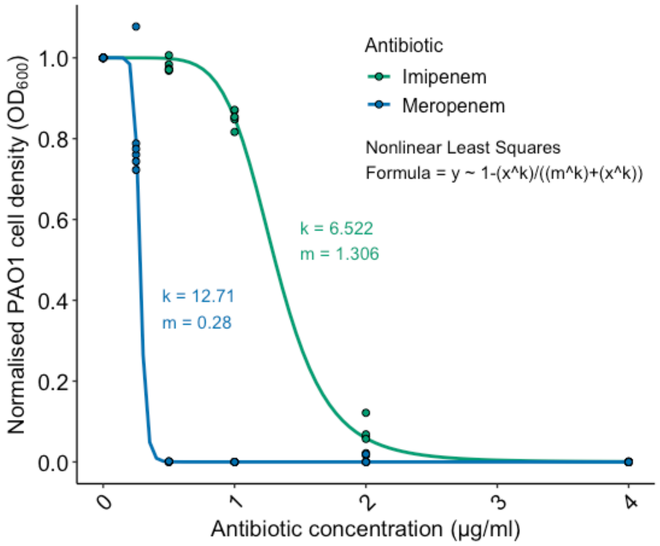

Fig. S6 Comparison between PAO1 meropenem and imipenem dose-response MIC curves. Nonlinear least squares fitting of the hill function *y ~ 1-(x^k)/((m^k)+(x^k))* was conducted using {nls} function in R. MIC Data from Fig. 1a and Fig. 3a in the main text plotted on a linear x scale. All OD values normalised to the max growth in the absence of antibiotic. Estimated parameters for meropenem: k = 12.71, m = 0.28, and imipenem: k = 6.52, m = 1.31.

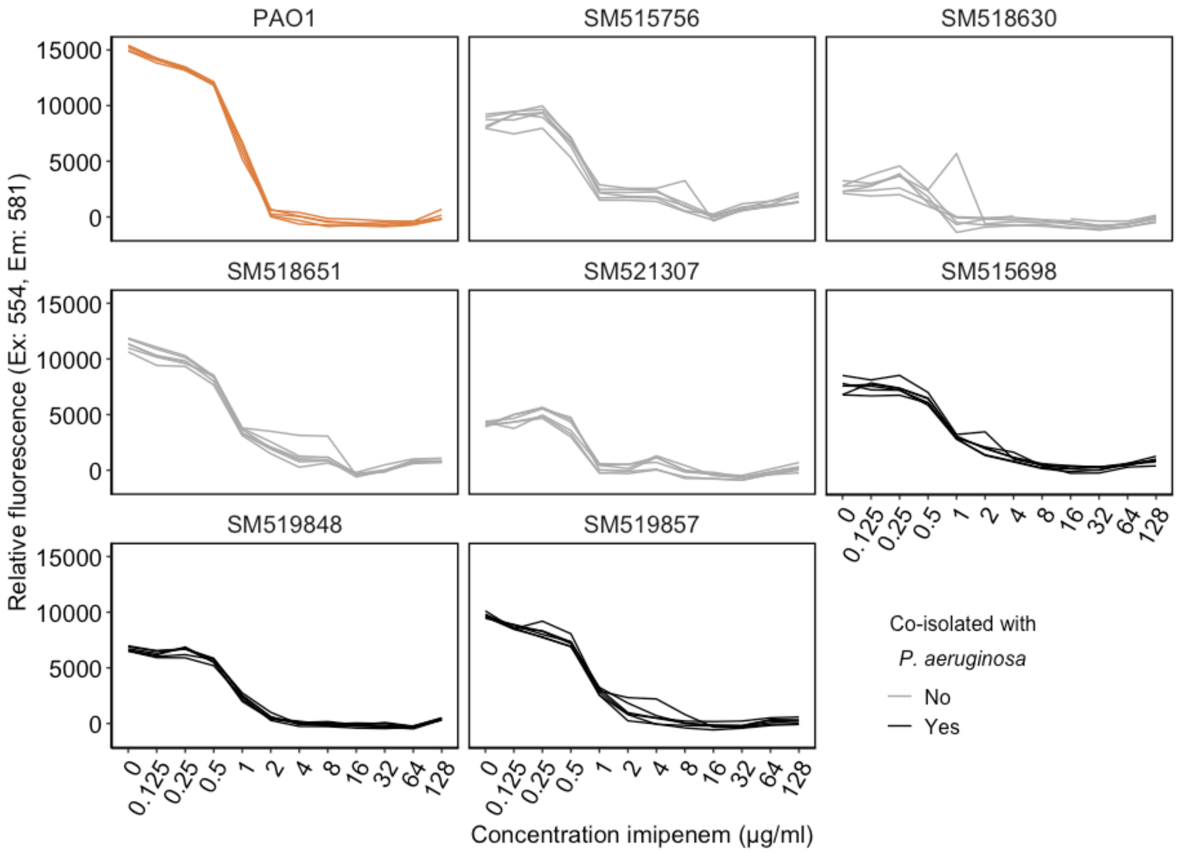

Fig. S7 Raw growth data used to calculate MICs presented in Fig 5 of the main text. Growth of PAO1 chromosomally labelled with dTomato was detected by red fluorescence. The *S. maltophilia* isolates produce no detectable auto-fluorescence at Ex 554, Em 581 (Figure S9). Lines are coloured by whether *P. aeruginosa* was co-isolated along with the *S. maltophilia* isolate from sputum samples, grey no *P. aeruginosa* present, black *P. aeruginosa* co-isolated with *S. maltophilia*. Each of the 6 independent replicates are plotted as separate lines (5 replicates for PAO1 monoculture control, orange).

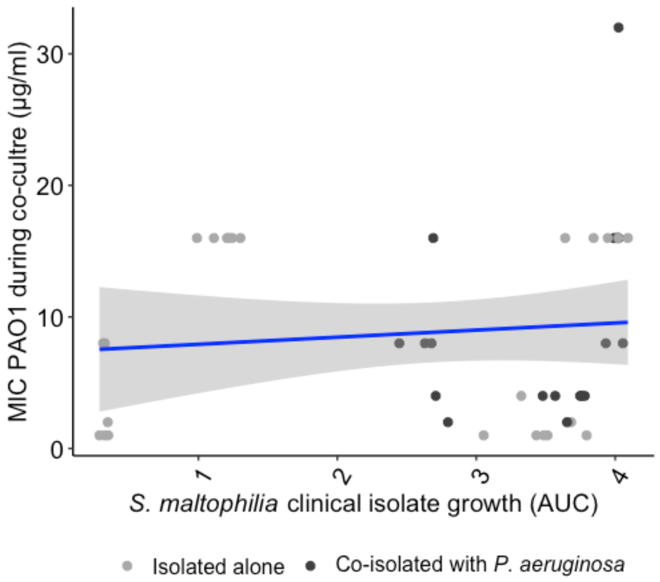

Fig. S8 The level of protection provided to PAO1 during coculture plotted against the ability of *S. maltophilia* to grow in the presence of imipenem as summarised by AUC of monoculture MIC curves presented in Fig. S9. There is no significant correlation between the two measures (Kendall's rank correlation, τ_b = 0.175, p = 0.133).

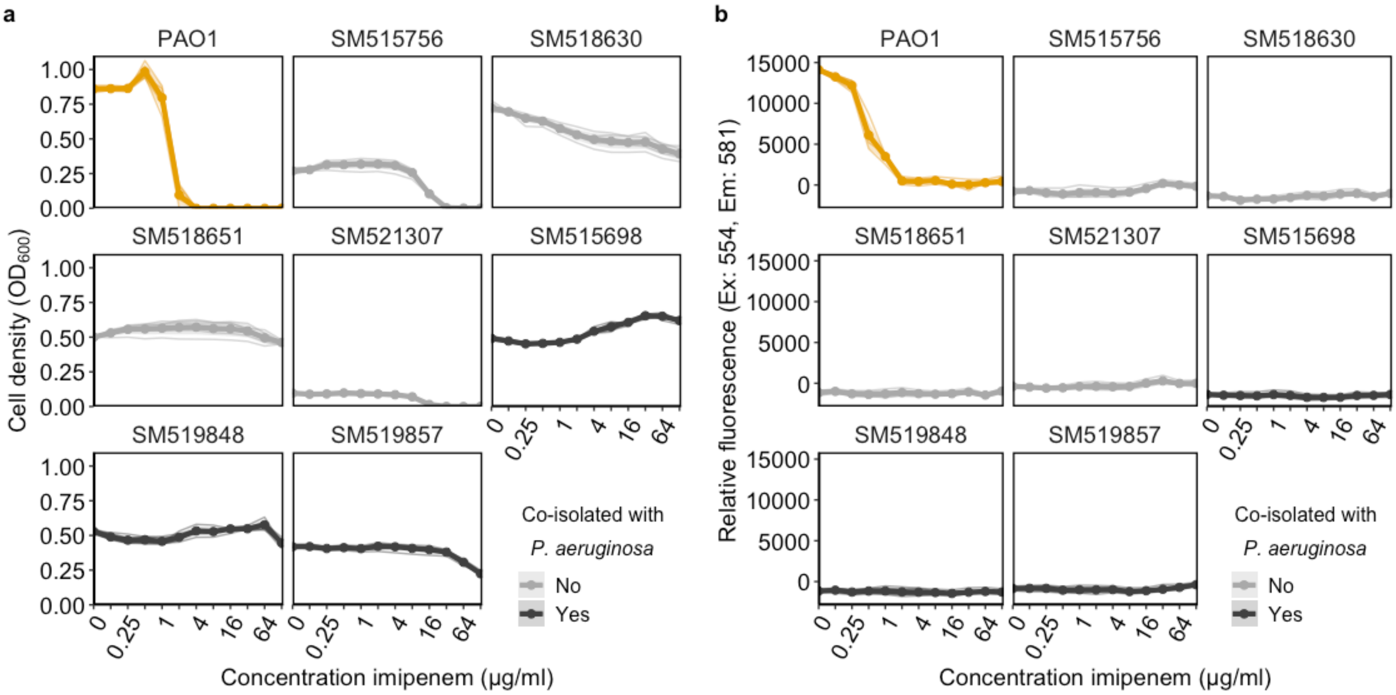

Fig. S9 a *S. maltophilia* CF clinical isolates MIC to imipenem, *S. maltophilia* plots are coloured by whether *P. aeruginosa* was co-isolated along with the *S. maltophilia* isolate from sputum samples, grey no *P. aeruginosa* present, black *P. aeruginosa* co-isolated with *S. maltophilia*. PAO1 control plotted in orange. Bold lines show mean of 6 biological replicates which are represented by narrow lines of the same colour. Shaded areas represent standard deviation. **b** Fluorescence measurements of monoculture MICs presented in panel **a**, none of the *S. maltophilia* isolates produce measurable autofluorescence. Bold lines show mean of 6 biological replicates which are represented by narrow lines of the same colour. Shaded areas represent standard deviation.

Table S1. Summary of clinical *S. maltophilia* CF isolates used in this study.

Strain ID	Isolation status	Growth in SCFM	MIC Imipenem μg/ml	*smeT-smeD* group	16S bp mismatches to K279a	BlaL1 identity to K279a
SM515756	Isolated alone	Y	32	A	2	99.0%
SM518651	Isolated alone	Y	>128	A	2	99.3%
SM518630	Isolated alone	Y	>128	C	7	87.8%
SM521307	Isolated with *S. aureus*	Y	16	A	2	No significant hit
SM515698	Isolated with *P. aeruginosa*	Y	>128	A	2	99.3%
SM517610	Isolated alone	N	Na	A	2	99.3%
SM519857	Isolated with *P. aeruginosa*	Y	>128	A	2	99.0%
SM519848	Isolated with *P. aeruginosa*	Y	>128	A	2	99.0%
SM519986	Isolated with *P. aeruginosa*	N	Na	A	2	99.0%

2 – ODE Modelling Supplementary Information

Model

We develop a simple ODE model to describe the kinetics of an antibiotic and the associated population dynamics of a mixed bacterial population containing a species sensitive to the antibiotic and a resistant species that inactivates the antibiotic. The purpose of the model is to elucidate the dynamics of exposure protection under different scenarios. In this way, the mechanisms driving the density dependent effect of exposure protection observed in Fig. 2 of the main text can be explained in a parsimonious way. In addition, the model provides hypotheses on the mechanisms driving differential protection provided by diverse *Stenotrophomonas maltophilia* isolates (Fig. 5 main text), and the differential protection provided against different antibiotics (Fig. 3 main text).

The differential equations describing the dynamics of sensitive species S, resistant species R, and antibiotic concentration A, are as follows:

$$\frac{dS}{dt} = \alpha_{\mathrm{max}} S\left(1 - \frac{S+R}{K}\right) - \mu_{\mathrm{ab}}(A)S - \mu_{\mathrm{bg}}S \qquad \text{(S1)}$$

$$\frac{dR}{dt} = \beta R\left(1 - \frac{S+R}{K}\right) - \mu_{\mathrm{bg}}R$$

$$\frac{dA}{dt} = -R\left(\frac{V_{\mathrm{max}}A}{K_{\mathrm{M}} + A}\right)$$

where parameters α_{max} and β are the growth rates of species S and R respectively, K is the local environmental carrying capacity, μ_{bg} is the background mortality rate, and $\mu_{\mathrm{ab}}(A)$ is the additional mortality rate caused to the S population by the antibiotic A. The concentration of antibiotic is reduced by the resistant population R according to Michaelis-Menten Kinetics parameterised by V_{max} the maximum hydrolysis rate achievable per cell and K_{M} the antibiotic concentration required to achieve half V_{max}.

If the antibiotic A dynamics are ignored (i.e. if $\frac{dA}{dt} = 0$ in (S1) and $\mu_{\mathrm{ab}}(A) = \mu_{\mathrm{ab}}$) then the model simply describes Lotka-Volterra competition between two bacterial species; the species with the larger ratio of growth rate to death rate (i.e. the ratio $\frac{\alpha_{\mathrm{max}}}{\mu_{\mathrm{ab}}+\mu_{\mathrm{bg}}}$ for S, and $\frac{\beta}{\mu_{\mathrm{bg}}}$ for R) will dominate and drive the other species to extinction. Because resistance is likely to

incur a cost, one might anticipate that $\alpha_{\max} > \beta$, and so the question of which species dominates is decided by magnitude of the additional mortality rate μ_{ab} suffered by the sensitive species S [1].

If the antibiotic A dynamics are now reintroduced (i.e. if the full model (S1) is considered) then the behaviour of the system depends on both the rate at which the resistant species R removes antibiotic A from the environment, and on the functional form of the additional antibiotic-induced mortality rate μ_{ab} affecting the S population. This dependence is discussed in some detail, below.

Initial versions of the model assumed that the death term due to antibiotic followed a standard $E_{\max}$ model [2, 3]:

$$\mu_{ab} = \frac{E_{\max} A^k}{{E_{50}}^k + A^k} \qquad \text{(S2)}$$

where $E_{\max}$ is the maximum effect of the antibiotic, E_{50} is the concentration of antibiotic required to achieve 50% $E_{\max}$, A is the current concentration of antibiotic and k is a Hill coefficient. Changes in k result in changes to the MIC, the concentration of antibiotic required to reduce net growth to zero. This makes it challenging to determine to what extent changes in MIC are due to the direct effects of changes in k, as opposed to changes in the protective effect of exposure protection due to the changes in slope of the sigmodal dose response curve.

Therefore instead of using the $E_{\max}$, the model uses a minimum net growth rate model of mortality proposed by Regoes *et al.* [4] which is parameterised using the MIC, $\alpha_{\max}$ the max achievable growth rate in the absence of antibiotic, $\alpha_{\min}$ the minimum net growth rate in the presence of antibiotic, A the concentration of antibiotic, and the Hill coefficient k:

$$\mu_{ab} = \frac{(\alpha_{\max} - \alpha_{\min})(A/MIC)^k}{(A/MIC)^k - \alpha_{\max}/\alpha_{\min}} \qquad \text{(S3)}$$

This way the slope of the sigmoidal death function can be altered without changing the MIC of the sensitive population (Fig. S10). Therefore, any changes in a species' ability to grow in the presence of antibiotic during coculture when changing the Hill function are due to the steepness of the sigmoidal relationship between A and μ_{ab}, altering exposure protection dynamics, rather than being due to implicit changes in MIC:

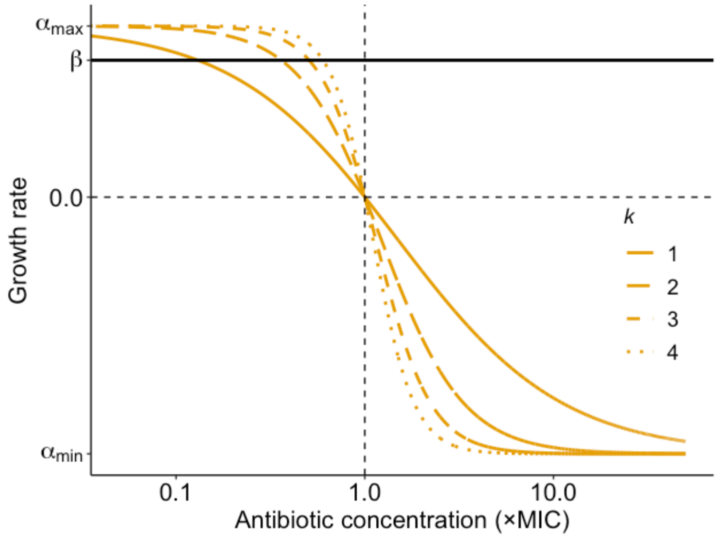

Fig. S10 Growth rate as a function of antibiotic concentration. Orange lines show growth rate of sensitive species with increasing Hill coefficient k, black line shows growth rate of resistant species. Figure adapted from Regoes *et al* [4].

Note that the definition of MIC used in equation (S3) differs slightly from the conventional definition of MIC. Conventionally MIC within the laboratory is defined as the concentration of antibiotic required to inhibit visible growth in an overnight culture. Here we describe the MIC as the concentration of antibiotic required to reduce the net growth rate of a population to zero.

Although the model's emergent dynamics arise essentially for the aforementioned Lotka-Volterra competition interaction, the full model as defined by equations (S1) and (S3) is highly nonlinear and is more readily explored via numerical integration. The results in Fig S11-S14 are produced numerical integration in R(3.6.1) using the LSOFA method within the {*deSolve*} package [5].

Results

Within monoculture the MIC of the sensitive population is defined by equation (S3), and results in net growth of 0 at the MIC of A (Fig S11), with concentrations greater than the MIC resulting in reductions in the S popultion. During coculture, species R inactivates the antibiotic allowing the sensitive species to grow in environments that otherwise would inhibit growth or be lethal (Fig. S11). At lethal concentrations of antibiotic (>MIC) there is an initial reduction in density of S, however once the environment is sufficiently detoxified growth

resumes. High concentrations of antibiotic reduce growth sufficiently to prevent the subsequent growth beyond the initial inoculum size.

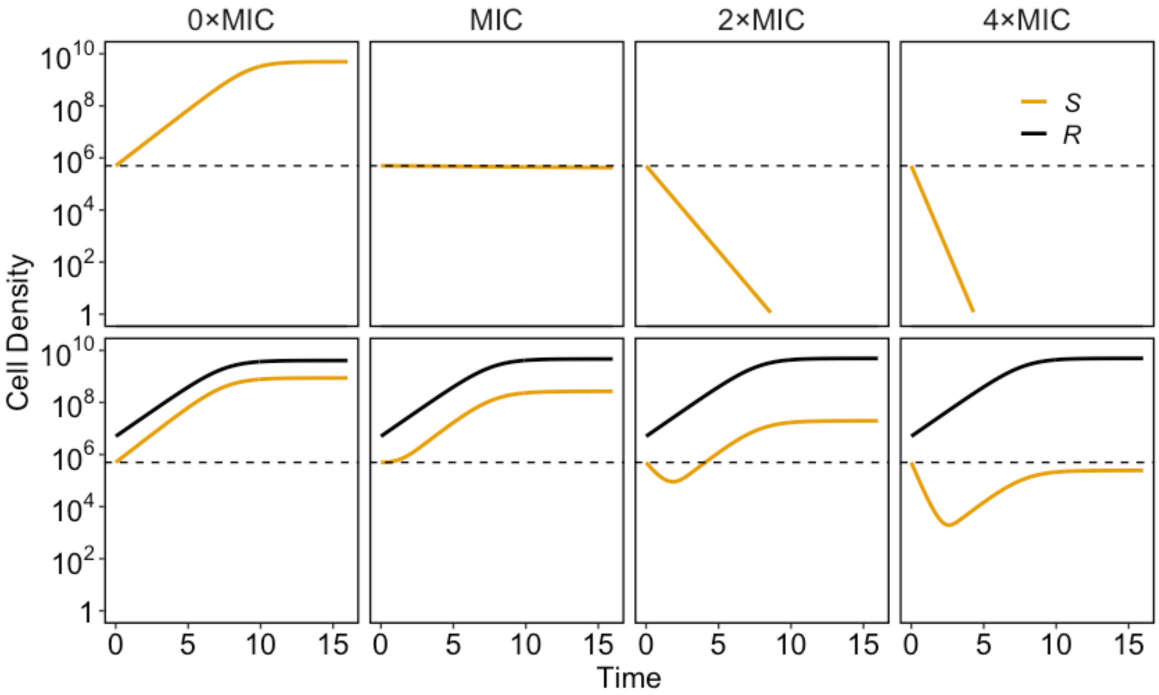

Fig. S11 The growth dynamics in mono and coculture. Top row shows the growth dynamics of the sensitive species alone in 0×MIC, MIC, 2×MIC and 8×MIC. The bottom row shows growth dynamics of two species cocultures consisting of sensitive species S (orange) and resistance species R (black). The horizontal dashed line shows the initial density of S, 5×10^5, the starting density of R is 5×10^6. α_{min} = -4, k = 2, V_{max} = 5×10^{-7}, K_{M} = 14, for other parameters see Table 1.

This exposure protection acts to increase the measure MIC of the sensitive species in coculture, as shown in Figure S12.

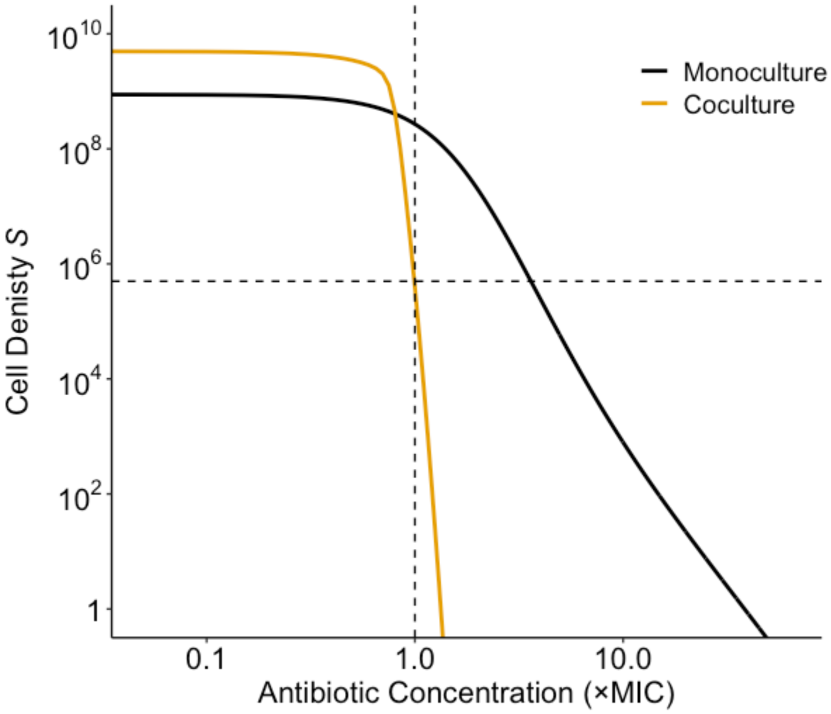

Fig. S12 End point density of S following 24 hours growth in monoculture (orange line) and coculture (black line). Horizontal dashed line shows the initial density of S. Coculture with R increases the realised MIC of S, intercept of black line with horizontal dashed line. Initial R = 5×10^6, α_{min} = -4, k = 2, V_{max} = 5×10^{-7}, K_{M} = 14, for other parameters see Table 1.

The model captures the effect of initial density; as the initial density of the resistant species increases the level of exposure protection provided to the sensitive species increases, this results in an increase in the realised MIC of the sensitive species in coculture (Fig. S13). With higher initial density of the resistant population the total net antibiotic inactivation rate increase. The increase in protection is prominent above initial R of 5×10^5. However, very low initial densities of R, between 5×10^0 and 5×10^4, do result in a small increase to the MIC of S. This is in contrast to the results presented in Figure 2 of the main text, where no increase in MIC was observed at or below an initial *S. maltophilia* density of 5×10^4 CFU/ml during the laboratory experiments. Importantly, the increase in MIC of S between 5×10^0 and 5×10^4 in the model were all below 2× the MIC of S in monoculture, a difference in MIC that was below the threshold of detection in the laboratory experiments as MIC was measured using a $\log_2$ dilution series. In addition, it has previously been shown that stochastic effects dominate the successful growth of bacteria when inoculated at such low densities [6], an effect that is not represented in this deterministic model.

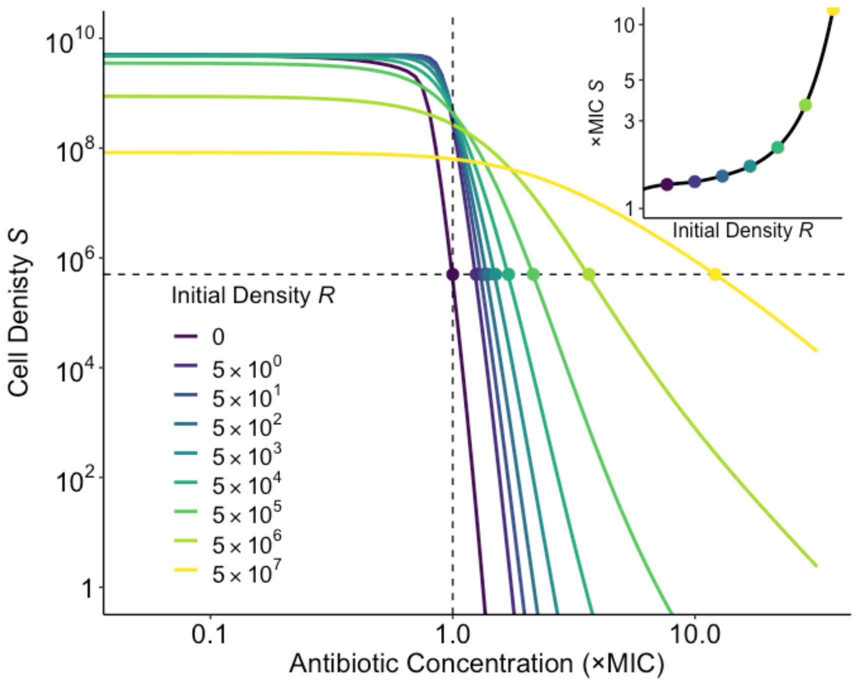

Fig. S13 Initial density of R determines the extent of exposure protection provided to S. A plot of the cell density of S as a function of antibiotic concentration while in coculture with R. Each line shows the endpoint density of S within independent sets of simulations with increasing density of R. Insert summarises the change in measured MIC within increasing initial density of R. Initial density S = 5×10^5, α_{min} = -4, k = 2, V_{max} = 5×10^{-7}, K_{M} = 14, for other parameters see Table 1.

It has previously been shown that high values of Hill coefficient k and maximum effect $(\alpha_{\text{max}} - \alpha_{\text{min}})$ are desirable properties for antimicrobials, as they help to reduce the probability that *de novo* resistance mutations emerge during treatment by reducing the size of the mutant selection window [7]. It is also likely that exposure protection will similarly be limited when the maximum effect (α_{min}) of the antibiotic and Hill coefficient (k) is high as the initial dose antibiotic will have a larger effect on the sensitive population at concentrations above the MIC (Fig. S10).

In addition to the sigmoidal relationship between growth and antibiotic concentration, the inactivation rate of the antibiotic plays a key role in the level of protection provided to the sensitive species. If the rate of antibiotic inactivation is too small, then the environment will not be detoxified quickly enough to allow the reinvasion of the sensitive species into the population following the initial death phase.

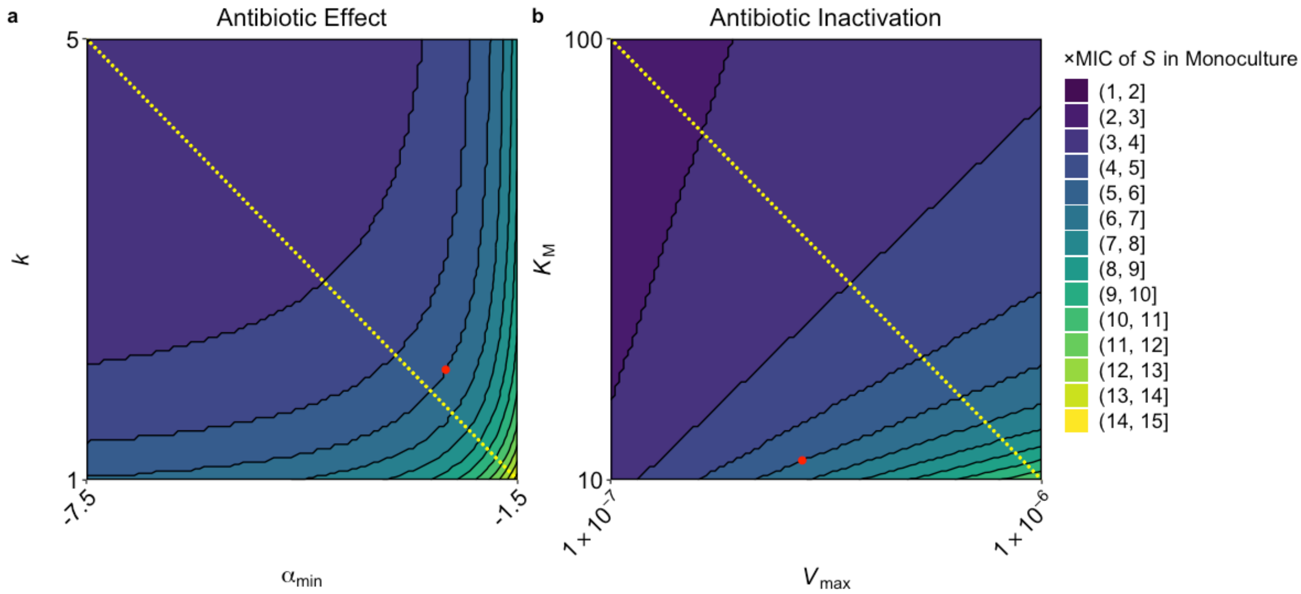

Fig. S14 Altering antibiotic efficacy and antibiotic inactivation parameters. **a** The effect of increasing $\alpha_{\min}$ and k on the level of protection provided to S during coculture. Increasing $\alpha_{\min}$ and k parameters fivefold 1 to 5, and -1.5 to -7.5 respectively. **b** The effect of increasing $V_{\max}$ and K_{M} on the level of protection provided to S during coculture. Increasing $V_{\max}$ and K_{M} parameters tenfold 1×10^{-7} to 1×10^{-6}, and 10 to 100 respectively. **a/b** Shading represents the level of protection provided to S as the ×MIC of S when in coculture compared to monoculture. Initial density R = 5×10^{6}, initial density S = 5×10^{5}, for other parameters see Table 1. Red point shows the $V_{\max}$ (5×10^{-7}) and K_{M} (14) used in **a** and $\alpha_{\min}$ (-2.5) and k (2) used in **b** for each respective set of simulations. Contours filled by the ×MIC of S in coculture, purple less protection, yellow more protection. Yellow points in panels **a** and **b** show the parameter values used in x and y axes respectively of Figure 4 in the main text.

Both increased bacterial killing and steeper dose response curves both act to reduce the level of exposure protection during coculture (Fig. S14a). It is important to note that changes in $\alpha_{\min}$ and k do not result in changes in the MIC of the sensitive isolate in monoculture. High values of $\alpha_{\min}$ or k alone are not sufficient to completely negate the protective effects of coculture with R. However, the combination between $\alpha_{\min}$ and k synergistically reduces protection (curved rather than linear response in the level of protection). Likewise, decreased antibiotic inactivation rate has a strong effect upon the level of exposure protection to S (Fig. S14b) with lower $V_{\max}$ or higher K_{M} values providing less protection. Therefore, less efficient β-lactamases, or lower β-lactamase expression, will result in lower

levels or protection to sensitive species and may help to explain the observed differences in protection between the clinical isolates of *S. maltophilia* presented in Fig. 5 of the main text.

Exposure protection strongly depends on both the rate of antibiotic inactivation and the shape of the dose response curve. Together, high effect antibiotics and low inactivation rates can almost completely negate the protective effects of antibiotic inactivation (Fig. S14). As meropenem has a steeper dose response curve in gram-negative bacteria, including *P. aeruginosa*, than imipenem [8], these results help to explain why no protection was observed to meropenem in coculture (Fig. 3b main text).

Table S2. Parameters used in the model.

Parameter	Description	Value	Unit
α_{max}	Maximum growth rate of S	1	h^{-1}
α_{min}	Minimum Growth rate of S in the presence of A	-1 ⟶ -5	h^{-1}
β	Growth rate of R	0.9	h^{-1}
S	Density of sensitive species	5×10^{5}	CFU/ml
R	Density of resistant species	0 ⟶ 5×10^{7}	CFU/ml
K	Carrying capacity	5×10^{9}	CFU/ml
MIC	Minimum inhibitory concentration required to reduce α to zero	2	µg/ml
k	Hill coefficient of antibiotic killing term	0.5 ⟶ 5.5	-
μ_{bg}	Background mortality rate	0.01	h^{-1}
A	Antibiotic concentration	0 ⟶ 30	×MIC
V_{max}	Maximum antibiotic inactivation rate	1×10^{-6} ⟶ 1×10^{-7}	µg/(h cell)
K_{M}	Antibiotic concentration to achieve half V_{max}	10 ⟶ 100	µg/ml

3 – References

1. Harrison E, Wood AJ, Dytham C, Pitchford JW, Truman J, Spiers A, et al. Bacteriophages Limit the existence conditions for conjugative plasmids. mBio. 2015;6:e00586-15.
2. Meibohm B, Derendorf H. Basic concepts of pharmacokinetic/pharmacodynamic (PK/PD) modelling. Int. J. Clin. Pharmacol. Ther. 1997;35:401–413.
3. Mouton JW, Dudley MN, Cars O, Derendorf H, Drusano GL. Standardization of pharmacokinetic/pharmacodynamic (PK/PD) terminology for anti-infective drugs: an update. J. Antimicrob. Chemother. 2005;55:601–607.
4. Regoes RR, Wiuff C, Zappala RM, Garner KN, Baquero F, Levin BR. Pharmacodynamic Functions: a Multiparameter Approach to the Design of Antibiotic Treatment Regimens. Antimicrob. Agents and Chemother. 2004;48:3670–3676.
5. Soetaert K, Petzoldt T, Setzer RW. Solving differential equations in R: Package deSolve. J. Stat. Softw. 2010;33:1–25.
6. Alexander HK, MacLean RC. Stochastic bacterial population dynamics restrict the establishment of antibiotic resistance from single cells. PNAS. 2020;117:19455–19464.
7. Yu Guozhi, Baeder Desiree Y., Regoes Roland R., Rolff Jens. Predicting drug resistance evolution: insights from antimicrobial peptides and antibiotics. Proc. Royal Soc. B. 2018;285:20172687.
8. Zhanel GG, Simor AE, Vercaigne L, Mandell L. Imipenem and meropenem: Comparison of in vitro activity, pharmacokinetics, clinical trials and adverse effects. Can. J. Infect. Dis. 1998;9:215–228.
